# Extracorporeal photopheresis reduces the T cell stimulatory capacity of human primary blood conventional dendritic cells type 1

**DOI:** 10.3389/fimmu.2025.1646421

**Published:** 2025-08-13

**Authors:** Lukas Heger, Carola Berking, Holger Hackstein

**Affiliations:** ^1^ Department of Transfusion Medicine and Hemostaseology, Universitätsklinikum Erlangen, Friedrich-Alexander-Universität Erlangen-Nürnberg, Erlangen, Germany; ^2^ Department of Dermatology, Universitätsklinikum Erlangen, Friedrich-Alexander-Universität Erlangen-Nürnberg, Erlangen, Germany; ^3^ Deutsches Zentrum Immuntherapie, Universitätsklinikum Erlangen, Friedrich-Alexander-Universität Erlangen-Nürnberg, Erlangen, Germany

**Keywords:** extracorporeal photopheresis, ECP, graft-versus-host disease, GvHD, dendritic cells, cDC1, apoptosis, T cells

## Abstract

**Introduction:**

Extracorporeal photopheresis (ECP) is an immunomodulatory treatment option for different T cell-mediated diseases such as cutaneous T cell lymphoma (CTCL) and chronic graft-versus-host disease (GvHD). While in CTCL the polarization of T cells is shifted towards T helper cells type 1 (TH1) and an immune response against the lymphoma is induced, ECP in GvHD rather leads to the expansion of regulatory T cells (Treg). How ECP regulates the immune response dependent on the underlying disease is still not exactly known. As dendritic cells (DCs) are crucial regulators of the immune response, it is supposed that they are key players in the immunomodulatory effects of ECP. However, due to the scarcity of primary DCs in blood, research has focused on *in vitro*-generated monocyte-derived DCs so far.

**Methods:**

Here, we present for the first time how the primary human blood DC subpopulations, i.e., conventional DCs type 1 (cDC1), cDC2, DC3, and plasmacytoid DCs (pDC), directly isolated from blood of healthy donors, respond to *in vitro* ECP treatment.

**Results:**

We demonstrate that the exposure to 8-methoxypsoralen and UV-A light irradiation induces apoptosis in Toll-like receptor ligand-activated cDC1 and pDC as well as - to a minor extent - in steady state cDC1, cDC2, and DC3. However, the selective effect of ECP on viability of DC subpopulations was dependent on culture duration (18h vs. 42h) as well as condition (steady state vs. TLR ligand activated). Further, ECP modulates the expression of the co-stimulatory and co-regulatory molecules CD40, CD86, and PD-L1 on DC subpopulations. While ECP did not affect the T cell stimulatory capacity of cDC2 and DC3, ECP-treated cDC1 and - to a minor extent - pDC showed reduced activation of memory T cells and diminished secretion of TH1- and TH17-associated cytokines.

**Conclusion:**

Thus, especially blood cDC1 are direct targets of ECP and the reduction of their T cell stimulatory capacity might contribute to the clinical efficacy observed in chronic GvHD patients.

## 1 Introduction

Extracorporeal photopheresis (ECP) is an immunomodulatory treatment for different T cell-mediated diseases. For the treatment, leukocytes of the patient are collected by apheresis, incubated with 8-methoxypsoralen (8-MOP), a photoactivatable substance, and exposed to UV-A irradiation followed by reinfusion of the treated leukocytes into the patient. Typically, patients are treated on two consecutive days with ECP in cycles every two to six weeks dependent on the severity of the symptoms. Mechanistically, UV-A light-activated 8-MOP induces single strand breaks in the DNA of the cells by forming adducts with pyrimidine bases of the DNA (1). Subsequently, the leukocytes undergo apoptosis when the repair mechanism of the cells are overloaded (1). ECP was first discovered in the 1980s to be effective for the treatment of cutaneous T cell lymphoma (CTCL) but is now also used to treat other T cell-mediated diseases, such as chronic graft-versus-host disease (GvHD) and chronic allograft rejection (2–5). In addition, ECP has been demonstrated to be effective as prophylactic treatment for the prevention of acute cellular rejection in lung allograft patients (6) and in reducing immune-related adverse events in cancer patients treated with immune checkpoint inhibitors (7, 8).

However, despite its broad use and effectiveness, the exact mechanism how ECP leads to immunomodulation is unclear and seems to depend on the underlying disease. In CTCL, ECP induces a shift from an IL-4-driven T helper cell (TH) type 2 environment to an IFNγ/IL-12-mediated TH1 immune response against the lymphoma (9). In contrast, murine models of GvHD showed an increase in regulatory T cells (Treg) in response to ECP treatment (10). Polarization of T cells into certain subtypes, such as TH1, TH2, or Treg, is usually regulated by dendritic cells (DCs) (11). DCs belong to the group of antigen-presenting cells (APCs) and are the most efficient cell type in inducing naïve T cell responses. DCs are present in both lymphoid as well as non-lymphoid tissues and are equipped with pattern recognition receptors, such as Toll-like receptors (TLRs), C-type lectin receptors, and NOD-like receptors, to sense the environment for pathogen- and danger-associated molecular patterns (PAMPs and DAMPs) (12). After the uptake of antigens by pinocytosis, phagocytosis, or receptor-mediated endocytosis, DCs process the antigens and present the fragments on the cell surface as peptide-MHC (major histocompatibility complex) complexes to T cells (11). Dependent on the environment, DCs either induce tolerance in steady state or T cell immunity under inflammatory conditions. In human and mice, several DC subpopulations exist that can be differentiated based on ontogeny, function, and surface marker expression (11). Conventional DC type 1 (cDC1) depend on the transcription factors IRF8 and BATF3 and human cDC1 can be identified by the expression of XCR1, CLEC9A, and CD141 (13–16). cDC1 excel in the cross-presentation of cell-associated antigens and, therefore, play a crucial role in anti-tumor immunity (17–22). cDC2 partly depend on the transcription factor IRF4 and human cDC2 are characterized by the expression of CD1c, FcϵR1A, and CLEC10A and also termed CD1c^+^ DCs (22–25). CD1c^+^ DCs are superior in the induction of T helper cell responses and can be further divided into bona fide CD5^+^ cDC2 with higher T cell-stimulatory capacity and monocyte-related CD163^+^CD64^+^ DC3 with a more pro-inflammatory phenotype (18, 26–32). In addition, plasmacytoid DCs (pDC) exist that rely on transcription factors such as E2–2 and SpiB and are identified in humans by the expression of CD123 and CD303 (33–36). pDC are known for their capacity to secrete high amounts of type I interferons and, thus, for their role in antiviral immunity (37, 38).

Due to their important role in the regulation of T cell immunity, ECP might influence DCs to either induce immunity in CTCL or tolerance in GvHD. However, as primary DCs are very rare cell types, accounting for less than 1% of peripheral blood mononuclear cells (PBMCs), much of the research has focused on monocyte-derived DCs (moDCs) or bone marrow-derived DCs (BMDCs), which can be generated in large quantities *in vitro* by culturing purified monocytes or bone marrow cells, respectively, in the presence of GM-CSF and IL-4 (39, 40). moDCs treated *in vitro* with ECP showed strong induction of apoptosis in the steady state as well as under inflammatory conditions (41). Further, ECP prevented the expression of co-stimulatory molecules as well as the secretion of IL-12 leading to a reduced induction of naïve T cell responses (41). When untreated moDCs were co-cultured with ECP-treated apoptotic lymphocytes, the moDCs showed reduced expression of co-stimulatory molecules as well as enhanced secretion of the anti-inflammatory cytokine IL-10 (42). Similarly, rat BMDCs showed a decreased expression of co-stimulatory molecules, an increased secretion of IL-10, and a reduced capacity to induce naïve T cell responses, when co-cultured with ECP-treated splenic cells (43). Thus, *in vitro*-generated BMDCs and moDCs seem to respond to ECP. However, moDCs do not reflect the primary blood DCs present in the apheresate of patients but rather correspond to inflammatory DCs (44).

Thus, data on the response of human primary blood DCs to ECP are still largely missing. Since infusion of ECP-treated enriched DCs was sufficient to transfer the therapeutic effect of ECP in a murine model of contact hypersensitivity (45), we investigated how human primary blood DCs respond to *in vitro* treatment with ECP. For this purpose, cDC1, cDC2, DC3, and pDC were isolated from the blood of healthy donors by cell sorting and treated with 8-MOP and UV-A light in steady state and in presence of TLR ligands. Subsequently, the induction of cell death, the expression of co-stimulatory and co-inhibitory molecules, cytokine secretion as well as the capacity to activate memory T cell responses were determined. We demonstrate that *in vitro* ECP induces apoptosis preferentially in cDC1 and pDC after stimulation with TLR ligands and strongly reduces the capacity of cDC1 to activate CD4^+^ and CD8^+^ memory T cell responses. Thus, in an inflammatory setting as observed in GvHD patients, the reduction of the T cell stimulatory capacity of cDC1 might contribute to the effector mechanism of ECP.

## 2 Materials and methods

### 2.1 Isolation of peripheral blood mononuclear cells from healthy blood donors

Leukocyte reduction system (LRS) cones and thrombocyte apheresis cassettes (TACs) were retrieved from healthy adults undergoing thrombocytapheresis at the Department of Transfusion Medicine and Hemostaseology of the University Hospital Erlangen. This study was performed with the informed written consents of all donors in accordance with the Declaration of Helsinki (approved by the local ethics committee [Ethikkommission der Friedrich-Alexander-Universität Erlangen-Nürnberg]; ethics vote 346_18 B). Peripheral blood mononuclear cells (PBMCs) were isolated from LRS cones and TACs as described before (46, 47). Briefly, the blood product was extracted and diluted with PBS. Subsequently, 20 ml of diluted blood product was overlaid onto 14 ml of Lymphocyte Separation Medium (Anprotec) and centrifuged for 20 min with 520 x g at room temperature without brakes. Then, the interphase containing the mononuclear cells was transferred to a 50 ml tube and washed twice with phosphate-buffered saline (PBS). After washing, cell numbers were determined using a Luna-FL Automated Fluorescence Cell Counter (Logos Biosystems) and the cells used for the experiments.

### 2.2 Cell sorting of human primary dendritic cell subpopulations

**Figure 1:**
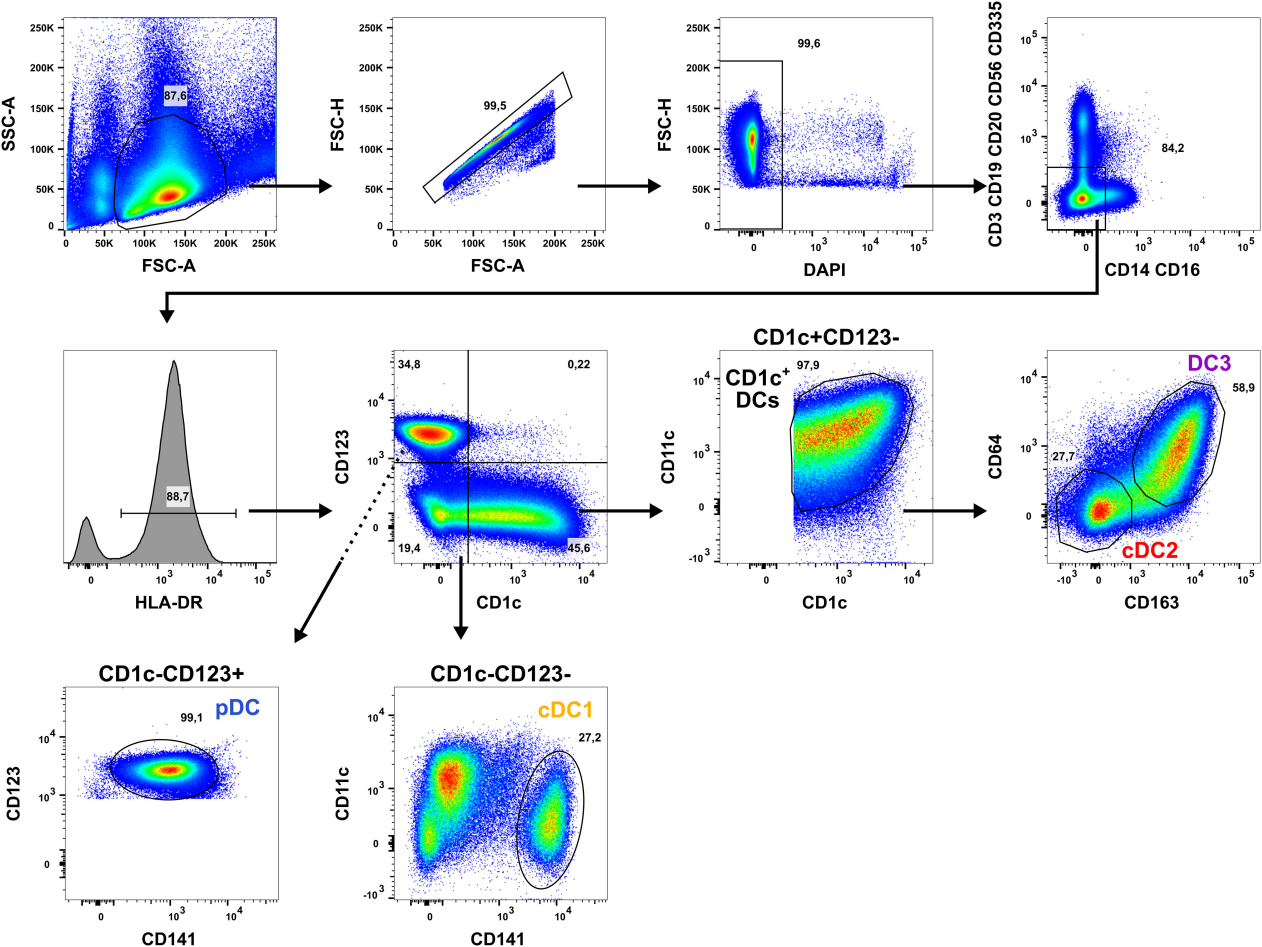
Applied gating strategy for the isolation of human DC subpopulations. PBMCs of healthy human adults undergoing thrombocytapheresis were enriched using the EasySep Pan-DC Pre-Enrichment kit and stained with a panel of fluorochrome-conjugated antibodies (**Table 1**). Then, DC subpopulations were sorted using a BD Aria II by gating for the morphology of leukocytes (FSC-A/SSC-A), singlets (FSC-A/FSH-H), and living cells (DAPI^-^). After exclusion of T cells (CD3^+^), B cells (CD19/CD20^+^), NK cells (CD56/CD335^+^), and monocytes (CD14/CD16^+^), DCs were selected by gating for HLA-DR^+^ cells. DCs were divided into CD1c^+^CD123^-^, CD1c^-^CD123^-^, and CD1c^-^CD123^+^ cells using a quadrant gate. CD1c^+^ DCs were identified in CD1c^+^CD123^-^ cells by co-expression of CD1c and CD11c and sorted into CD64^-^CD163^-^ cDC2 (red) and CD64^+^CD163^+^ DC3 (purple). cDC1 were gated in the CD1c^-^CD123^-^ fraction as CD141^+^CD11c^int^ cells (yellow-orange). In the CD1c^-^CD123^+^ quadrant, pDC were sorted as CD123^+^CD141^int^ cells. One representative donor is shown.

For experiments with human dendritic cell (DC) subpopulations, DCs were isolated from PBMCs of healthy adults by cell sorting as described before (16, 31, 46). Briefly, PBMCs were resuspended in PBS + 2% fetal calf serum (FCS) + 1 mM EDTA (EasySep Buffer) with a concentration of 1 x 10^8^ cells/ml. Up to 9 ml of cell suspension were transferred to 14 ml roundbottom tubes and enriched using the EasySep Human Pan-DC Pre-Enrichment Kit (Stemcell Technologies). Subsequently, enriched DCs were stained with a panel of fluorochrome-coupled antibodies and stained on ice for 30 min. After washing, cells were resuspended in EasySep Buffer + DAPI (100 ng/ml) and sorted into sterile FACS tubes using a BD FACSAria II cell sorter with a 70 µm nozzle. DCs were defined as Lin^-^ (CD3/CD19/CD20/CD56 and CD14/CD16) and sorted as cDC1 (HLA-DR^+^CD141^+^CD11c^int^CD1c^-^CD123^-^), cDC2 (HLA-DR^+^CD1c^+^CD11c^+^CD64^-^CD163^-^CD123^-^), DC3 (HLA-DR^+^CD1c^+^CD11c^+^CD64^+^CD163^+^CD123^-^), and pDC (HLA-DR^+^CD123^+^CD141^int^CD1c^-^CD11c^-^) as shown in **Figure 1**.

### 2.3 *In vitro* treatment with extracorporeal photopheresis

For *in vitro* treatment with ECP, sorted human primary DC subpopulations were resuspended in DC medium (RPMI-1640 + 10% human serum type AB + 1% L-Glutamax + 1% Penicillin/Streptomycin + 1% Na-Pyruvat + 1% non-essential amino acids + 1% HEPES) and seeded in sterile 96-well plates (V-bottom). Then, either 400 ng/ml 8-MOP or solvent control (equal volume ethanol) were added and the cells incubated at 37°C for 30 min. After incubation, plates were either irradiated with 2 J/cm² UV-A light (BIO-LINK BLX-365 irradiation chamber) or MOCK treated (48). After centrifugation for 10 min at room temperature with 300 x g, supernatant was removed and the cells resuspended either in DC medium (steady state) or in DC medium + 5 µg/ml R848, 5 µg/ml pIC, 100 ng/ml CRX-527, or 2.5 µM CpG (ODN2216) (inflammatory conditions). Cells were cultured for different time points (18 h, 42 h) before flow cytometric analysis and harvesting of supernatants for cytometric bead assay (CBA) analysis. For flow cytometric analysis, cells were stained with BV605-coupled anti-CD1c (clone: L161, BioLegend), PE/Cy7-coupled anti-CD11c (clone: 3.9, BioLegend), A647-coupled anti-CD64 (clone: 10.1, BioLegend), BV510-coupled anti-CD123 (clone: 6H6, BioLegend), BV421-coupled anti-CD141 (clone: M80, BioLegend), PE/Dazzle 594-coupled anti-CD163 (clone: GHI/61, BioLegend), and APC/Cy7-coupled anti-HLA-DR (clone: L243, BioLegend) for identification of DC subpopulations. In addition, they were stained with either A700-coupled anti-CD40 (clone: 5C3, BioLegend), FITC-coupled anti-CD86 (clone: Bu63, BioLegend), BV650-coupled anti-PD-L1 (clone: 29E2A3, BioLegend) or respective isotype controls for 30 min on ice. After washing, cells were stained with PE-coupled Annexin V (BioLegend) and 7-AAD (BioLegend) for 20 min on ice. After washing, cells were acquired using a Cytoflex S (Beckman Coulter) and analyzed using FlowJo Software (V10). Supernatants of the cells were stored at -80°C until analysis for secreted cyto- and chemokines by CBA using the LEGENDplex Human Macrophage/Microglia Panel (BioLegend). Supernatants were thawed and concentrations of the cytokines IL-12p70, TNF-α, IL-6, IL-4, IL-10, IL-1β, Arginase, CCL17 (TARC), IL-1RA, IL-12p40, IL-23, IFN-γ, and CXCL10 (IP-10) determined as described by the manufacturer. Subsequently, samples were acquired using a Cytoflex S (Beckman Coulter) and analyzed using the LEGENDplex Data Analysis Software Suite (Qognit). Then, the data were normalized based on the highest measured value (median of six/five individual values for each condition) in the complete dataset for each cytokine and plotted as heatmap. The maximal measured median value was given as reference in the figure. Based on the color code (% of max. values) and the max. value measured in the dataset, the median concentration for each subset and condition can be determined.

### 2.4 Activation of antigen-specific memory T cells

In order to perform co-cultures of DC subpopulations and autologous memory T cells, cDC1, cDC2, DC3, and pDC were sorted from healthy blood donors as described above. Autologous memory T cells were isolated from the same blood donor by negative enrichment using the MojoSort Human CD3 T Cell Isolation Kit (BioLegend) with addition of biotinylated anti-CD45RA antibody (clone: HI100, BioLegend) to deplete naïve T cells. To measure proliferation of T cells, isolated memory T cells were labeled with 5 µM CFSE (BioLegend) for 15 min at 37°C prior to the co-culture.

Before the co-culture, DCs were incubated with 400 ng/ml 8-MOP or solvent control for 30 min at 37°C. Then, DCs were irradiated with 2 J/cm^2^ UV-A light (BIO-LINK BLX-365 irradiation chamber) or MOCK treated. After centrifugation for 8 min with 300 x g at 30°C, supernatant was removed and DCs resuspended in medium either with 1 nM CEFT peptides for presentation on MHC-I and MHC-II molecules (JPT petide technologies), 1 nM CEFT peptides + 1 µg/ml R848, or 10 CFU/DC heat-killed *E. coli* (Invivogen). After 18 h at 37°C, autologous CFSE^+^ memory T cells were added in a 1:10 DC:T cell ratio and co-cultured for five days. After the co-culture, supernatants were stored at -80°C until analysis for cytokine secretion using the LEGENDplex Hu Th Cytokine Panel (BioLegend). The T cells were analyzed by flow cytometry for proliferation (dilution of CFSE signal) as well as phenotype (activation and exhaustion markers). Therefore, cells were stained with BV510-coupled anti-CD3 (clone: OKT3, BioLegend), APC/Cy7-coupled anti-CD4 (clone: OKT4, BioLegend), PE/Cy5-coupled anti-CD8a (clone: HIT8a, BioLegend) as well as either APC-coupled anti-CD25 (clone: BC96, BioLegend), PE-coupled anti-CD71 (clone: CY1G4, BioLegend), A700-coupled anti-CD197 (clone: G043H7, BioLegend), BV605-coupled anti-CD223 (clone: 11C3C65, BioLegend), as well as PE/Cy7-coupled anti-CD178 (clone: NOK-1, BioLegend) or respective isotype controls for 30 min on ice. After washing, cells were stained with 100 ng/ml DAPI (Carl Roth) for 5 min on ice. Then, cells were acquired using a Cytoflex S (Beckman Coulter) and analyzed using FlowJo Software.

### 2.5 Statistical analysis

Statistical analysis was performed in GraphPad Prism (V10) using 2way ANOVA for grouped data with Dunnett’s multiple comparisons tests as posthoc test. Individual symbols were used for each donor. In figures showing data from the same timepoint, donors can be traced by these individual symbols (all Figures for 18h time point, all Figure for 42h time point as well as all Figures and all Supplementary Figures for memory T cell assay).

## 3 Results

### 3.1 Extracorporeal photopheresis induces apoptosis especially in cDC1 and pDCs stimulated with TLR ligands

**Figure 2:**
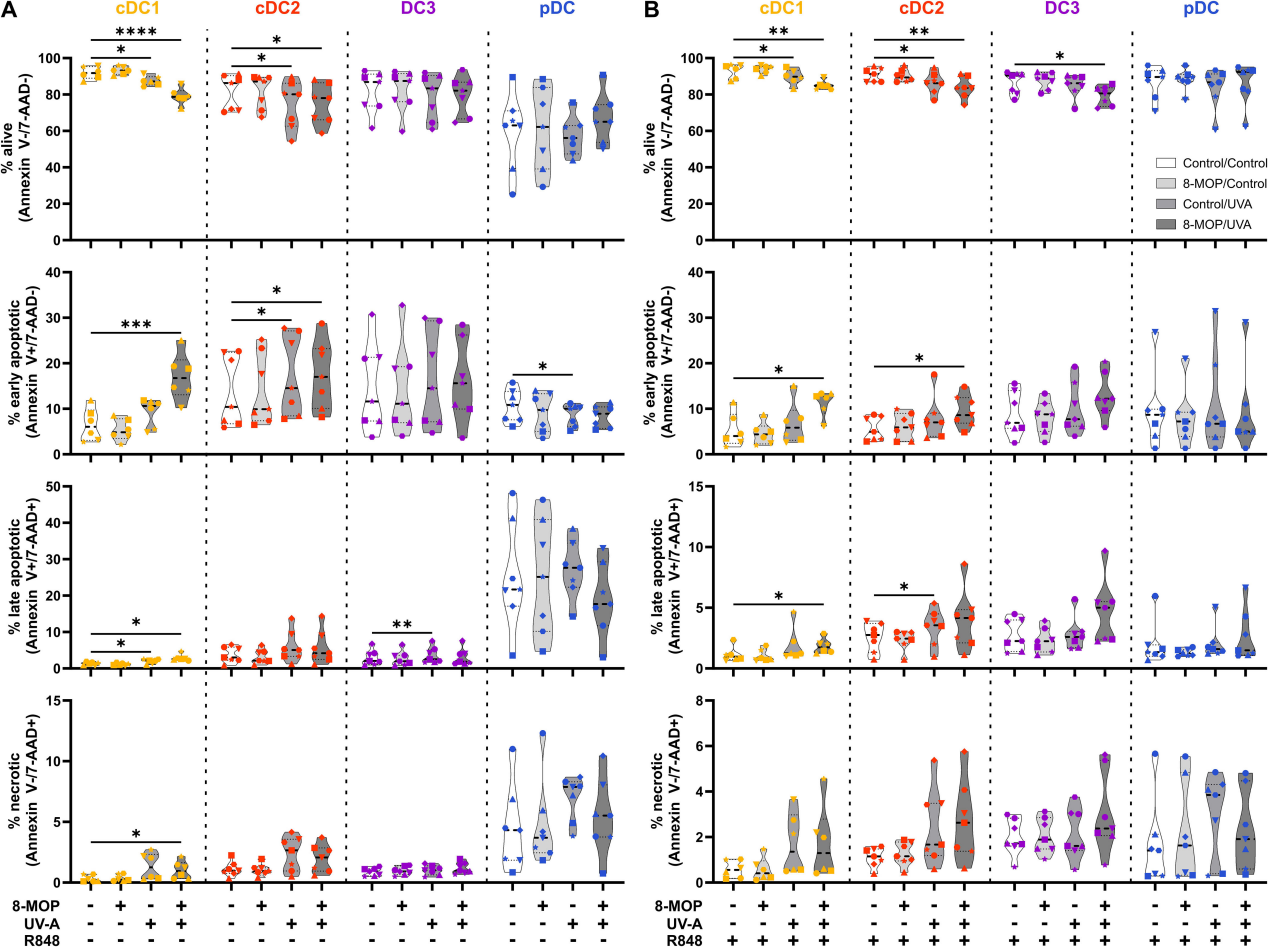
Experimental ECP induces apoptosis primarily in human blood cDC1 after 18 h of culture. Cell sorter-purified cDC1, cDC2, CD14^-^ DC3, and pDC were incubated either with 400 ng/ml 8-MOP or equal amount of solvent control (ethanol) for 30 min at 37°C as indicated below the figure. Then, cells were either irradiated with 2 J/cm^2^ UV-A light or mock-treated. After centrifugation to remove the solvent, cells were resuspended in **(A)** medium or **(B)** medium containing 5 µg/ml R848. After 18 h of culture, DCs were stained with the antibodies used for cell sorting and 7-AAD and Annexin V-PE to determine viability. Truncated violin plots depict percentages of alive (Annexin V^-^/7-AAD^-^), early apoptotic (Annexin V^+^/7-AAD^-^), late apoptotic (Annexin V^+^/7-AAD^+^), and necrotic (Annexin V^-^/7-AAD^+^) cDC1 (yellow-orange symbols), cDC2 (red symbols), DC3 (purple symbols) and pDC (blue symbols) of six donors (each donor with an individual symbol). Statistical analysis was performed in GraphPad Prism (V10) using 2way ANOVA for grouped data with Dunnett’s multiple comparisons tests as posthoc test (*p < 0.05, **p < 0.01, ***p < 0.001, ****p < 0.0001).

**Figure 3:**
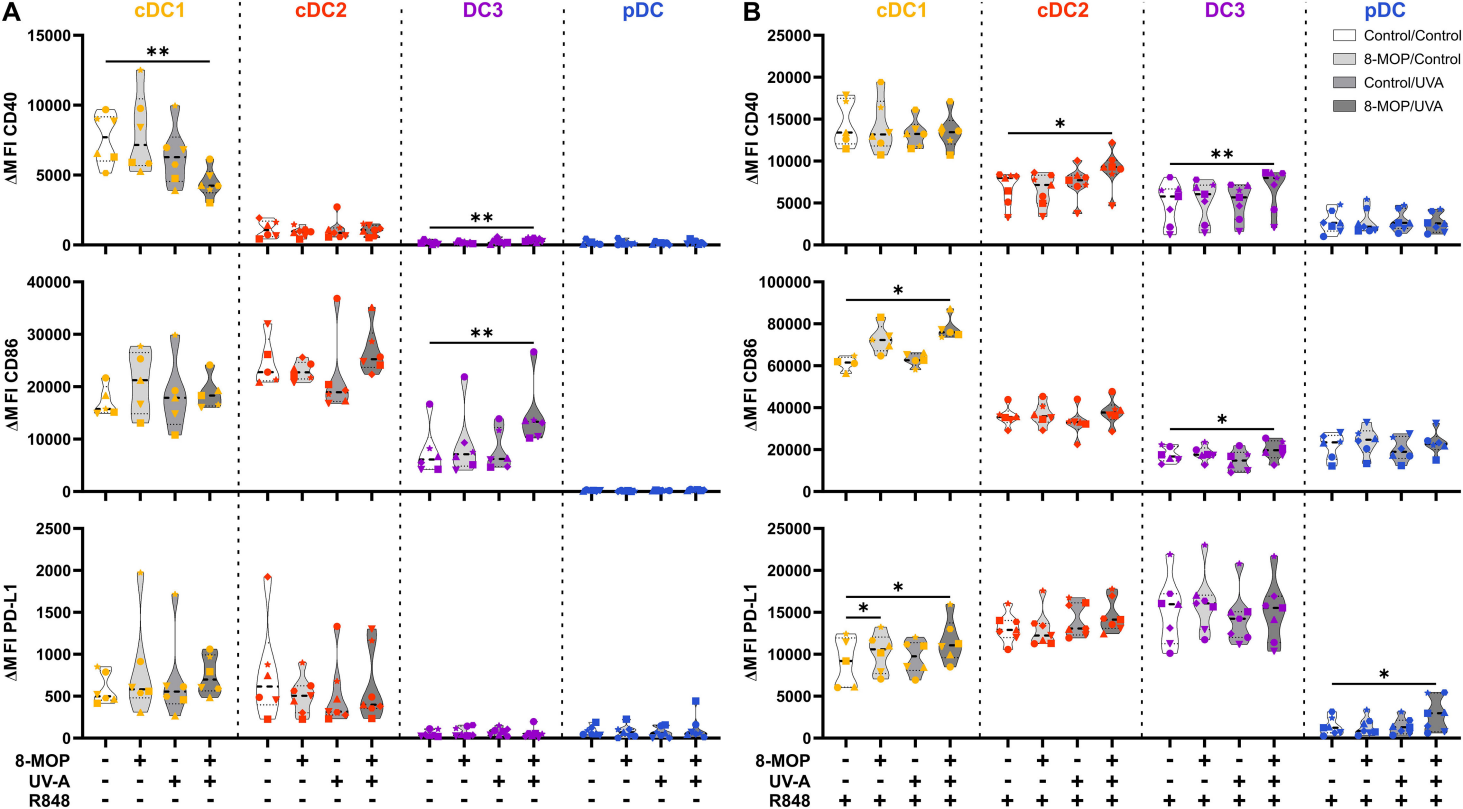
Experimental ECP modulates the expression of co-stimulatory and -regulatory molecules on human primary DCs after 18 h of culture. Cell sorter-purified cDC1, cDC2, CD14^-^ DC3, and pDC were incubated either with 400 ng/ml 8-MOP or equal amount of solvent control (ethanol) for 30 min at 37°C as indicated below the figure. Then, cells were either irradiated with 2 J/cm^2^ UV-A light or mock-treated. After centrifugation to remove the solvent, cells were resuspended in **(A)** medium or **(B)** medium containing 5 µg/ml R848. After 18 h of culture, DCs were stained with the antibodies used for cell sorting and A700-coupled anti-CD40, FITC-coupled anti-CD86, and BV650-coupled anti-PD-L1 or respective isotype controls. Truncated violin plots show ΔMFI on alive (Annexin V^-^/7-AAD^-^) cDC1 (yellow-orange symbols), cDC2 (red symbols), DC3 (purple symbols) and pDC (blue symbols) of six donors (each donor with an individual symbol). Statistical analysis was performed in GraphPad Prism (V10) using 2way ANOVA for grouped data with Dunnett’s multiple comparisons tests as posthoc test (*p < 0.05, **p < 0.01, ***p < 0.001, ****p < 0.0001).

ECP is an immunomodulatory treatment inducing apoptosis in lymphocytes as well as modulating the immune response towards TH1 in CTCL or Tregs in GvHD. In contact hypersensitivity in mice, ECP-treated DCs are sufficient to transfer the tolerogenic effect of ECP (45). However, it is unclear how primary human DCs, which are present in the photopheresate of patients, respond to the treatment with 8-MOP and UV-A light irradiation. As DCs are the main regulators of T cell responses, we speculated that ECP affects DCs directly. Due to the scarcity of DCs, we decided to perform *in vitro* ECP of sorted human primary blood DCs from healthy blood donors. Therefore, we enriched all human DC subpopulations from blood of healthy donors by negative enrichment followed by cell-sorting as described recently (31, 46). For cell sorting, enriched DCs were stained with a panel of fluorochrome-coupled antibodies (**Table 1**) and sorted into CD141^+^CD11c^int^ cDC1, CD1c^+^CD11c^+^CD64^-^CD163^-^ cDC2, CD1c^+^CD11c^+^CD14^-^CD64^+^CD163^+^ DC3 (CD14^-^ DC3), and CD123^+^CD141^int^CD11c^-^ pDC (**Figure 1**). When we cultured the DCs for 18h at 37°C, the phenotype of the DC subpopulations was stable: cDC1 showed expression of CD141 and CD11c but lacked markers of the DC2 lineage (**Supplementary Figure 1**). Both cDC2 and CD14^-^ DC3 remained CD1c^+^CD11c^+^. While they still could be differentiated based on CD163 and CD64 expression, the signal was weaker compared to freshly isolated DC3 (**Supplementary Figure 1**). Further, pDC could be identified by CD123 and CD141 expression as during cell sorting and did not express DC2-associated markers such as CD1c (**Supplementary Figure 1**). For the *in vitro* treatment with ECP, the purified DC subpopulations were incubated with 400 ng/ml 8-MOP or ethanol as solvent control for 30 minutes at 37°C followed by irradiation with 2 J/cm² UV-A or mock treatment. Subsequently, the medium containing 8-MOP or ethanol was replaced with fresh medium to mimic reinfusion of the photophoresate into the patients resulting in strong dilution of the photophoresate. The DCs were then either cultured in presence of the TLR ligand R848, as a model for DC activation in inflammatory conditions, or in medium to mimic steady state conditions. Since ECP induces apoptosis in lymphocytes such as T cells, we analyzed the induction of cell death using flow cytometry by staining the cells with Annexin V and 7-AAD to distinguish between early (Annexin V^+^/7-AAD^-^) and late apoptosis (Annexin V^+^/7-AAD^+^) as well as necrosis (Annexin V^-^/7-AAD^+^) (**Supplementary Figure 2**). After 18 h of culture, we observed induction of apoptosis in cDC1 and cDC2 in steady state conditions as well as in cDC1, cDC2, and CD14^-^ DC3 after TLR stimulation (**Figure 2**). Since ECP has been shown to influence the phenotype of immune cells such as monocytes (49, 50), we were interested in how *in vitro* ECP would modulate the expression of co-stimulatory and -regulatory molecules and the secretion of cyto- and chemokines in steady state as well as upon TLR stimulation. Therefore, we analyzed the living DCs (Annexin V^-^/7-AAD^-^) for the expression of co-stimulatory (CD40, CD86) and -regulatory (PD-L1) molecules by flow cytometry (**Supplementary Figure 3**), while the supernatants of the cells were collected for analysis of secreted cyto- and chemokines. After 18h, we observed only minor changes in the expression of co-stimulatory and -regulatory molecules (**Figure 3**). In steady state, 8-MOP/UV-A-treated cDC1 showed enhanced expression of CD40, whereas upon TLR stimulation CD86 and PD-L1 were enhanced on *in vitro* ECP-treated cDC1 (**Figure 3**). The phenotype of CD14^-^ DC3 was more pro-inflammatory under ECP conditions both in steady state (CD86) as well as after stimulation with R848 (CD40 and CD86), whereas ECP-treated pDC showed enhanced expression of the immunoregulatory molecule PD-L1 after TLR stimulation (**Figure 3**). The surface phenotype of cDC2 remained largely unchanged except for increased CD40 expression after ECP in presence of the TLR ligand R848 (**Figure 3**). In order to analyze whether these changes were specific to stimulation of TLR7/8, we used the TLR3 ligand pIC for cDC1, the TLR4 ligand CRX-527 for cDC2 and CD14^-^ DC3, and the TLR9 ligand CpG for pDC. Then, we analyzed the induction of cell death as well as co-stimulatory and -regulatory molecule expression as before. As with R848, we observed induction of apoptosis in cDC1 and CD14^-^ DC3 after TLR3 and TLR4 stimulation, respectively, whereas the cell death of cDC2 were rather dependent on UV-A light irradiation (**Supplementary Figure 4A**). Except for upregulation of PD-L1 on DC3 and pDC, we did not observe changes in the expression of co-stimulatory or -regulatory molecules (**Supplementary Figure 5A**).

**Table 1:** Antibody panel for cell sorting of human DCs.

Fluorochrome	Antigen	Clone	Vendor	Catalogue	Dilution
BV421	CD141	M80	BioLegend	344114	1:50
BV510	CD123	6H6	BioLegend	306022	1:50
BV605	CD1c	L161	BioLegend	331538	1:100
FITC	CD3	SK7	BioLegend	344804	1:200
	CD19	HIB19	BioLegend	302206	1:200
	CD20	2H7	BioLegend	302304	1:200
	CD56	HCD56	BioLegend	318304	1:200
	CD335	9E2	BioLegend	331922	1:200
PE/Dazzle 594	CD163	GHI/61	BioLegend	333624	1:100
PE/Cy7	CD11c	3.9	BioLegend	301608	1:100
A647	CD64	10.1	BioLegend	305012	1:100
A700	CD14	M5E2	BioLegend	301822	1:200
	CD16	3G8	BioLegend	302025	1:200
APC/Cy7	HLA-DR	L243	BioLegend	307618	1:100

**Figure 4:**
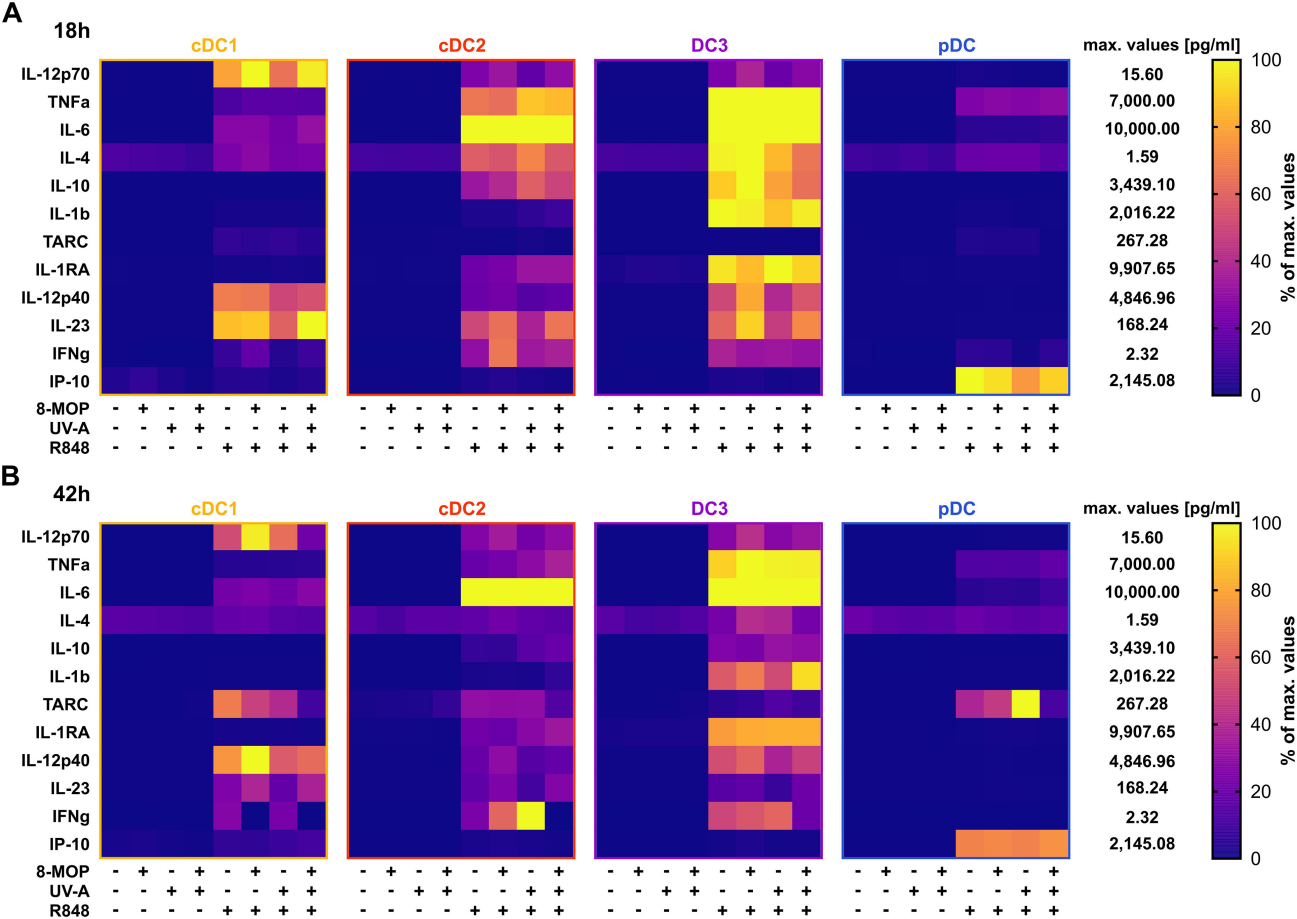
R848-induced cyto- and chemokine secretion by DCs is minorily influenced by *in vitro* treatment with ECP. Supernatants of DC subpopulations treated either with ECP (8-MOP/UV-A) or control conditions for **(A)** 18 h or **(B)** 42 h were analyzed by LEGENDplex Human Macrophage/Microglia Panel (BioLegend) for the secretion of cyto- and chemokines. Measured concentrations (pg/ml) were normalized based on the highest measured median value for each analyte in the whole data set. Percentage of maximum for cDC1, cDC2, CD14^-^ DC3, pDC is shown (each square shows the mean of **(A)** six or **(B)** five donors).

As ECP was reported to induce spontaneous release of IL-10 by myeloid CD1c^+^ DCs isolated from the photophoresate of refractory chronic GvHD patients (51), we were interested in how *in vitro* ECP would affect the secretion of cyto- and chemokines by sorted DCs. We determined the concentration of secreted cyto- and chemokines in the supernatants of the treated DCs by CBA using the LEGENDplex Human Macrophage/Microglia Panel. As expected, cDC1 were the main producers of IL-12 family members, i.e., IL-12p70, IL-12p40, and IL-23, after stimulation with R848, while cDC2 and CD14^-^ DC3 excelled in the secretion of the pro-inflammatory cytokines IL-6, TNFα, and IL-1β as well as the anti-inflammatory cytokines IL-10 and IL-1RA (**Figure 4A**). In contrast, pDC were the main producers of the chemokine CXCL10 (IP-10) that is dependent on signaling of interferon response factors (IRFs) (**Figure 4A**). When analyzing the impact of *in vitro* ECP, we did not observe induction of pro- or anti-inflammatory cytokines in steady state conditions and only slight changes on the secretion of cyto- and chemokines after TLR stimulation (cDC2: TNFα↑, IL-1β↑, IL10↑, IL-1RA↑; CD14^-^ DC3: IL-10↓; pDC: CXCL10↓; **Figure 4A**).

**Figure 5:**
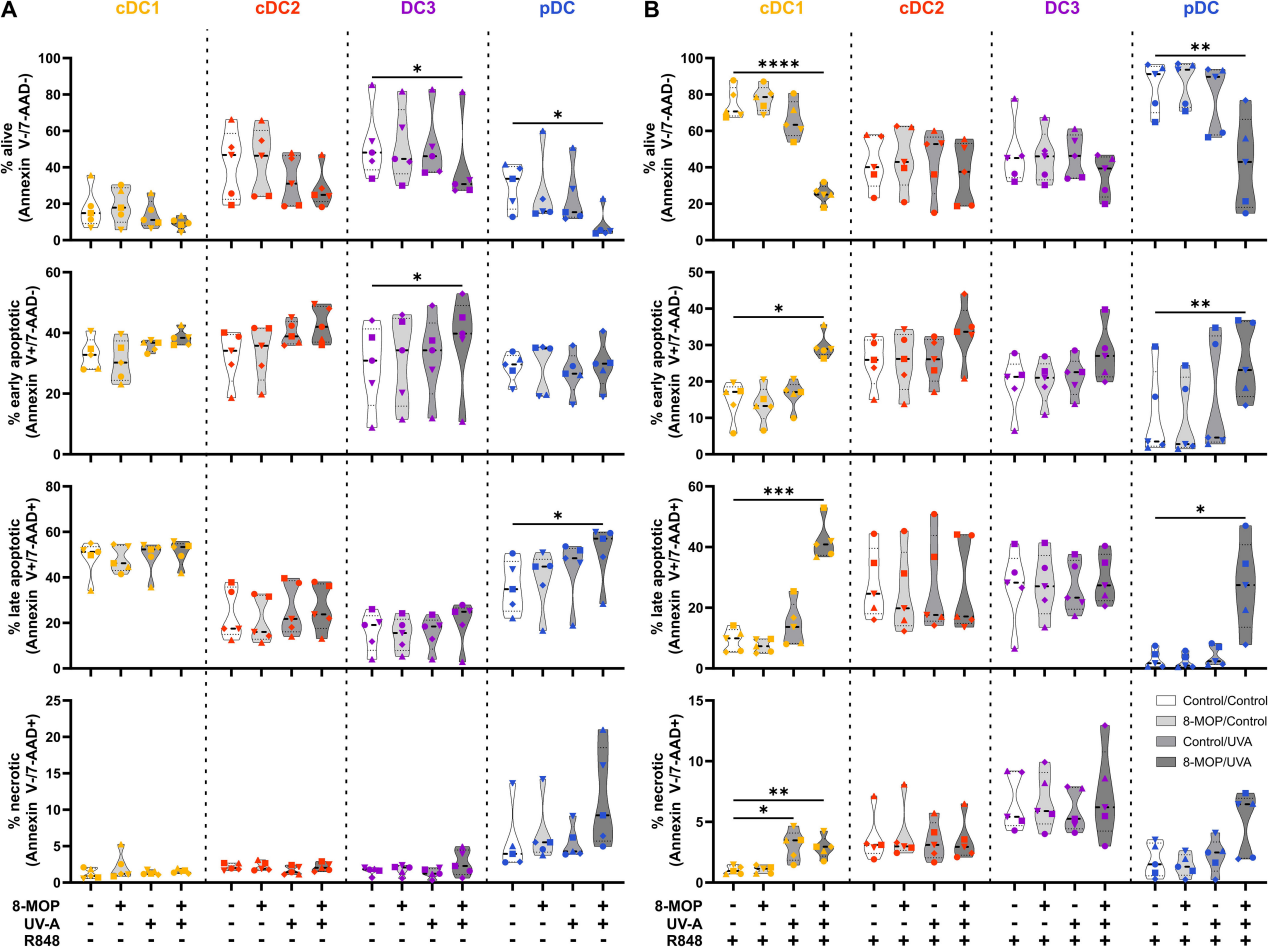
Experimental ECP induces apoptosis primarily in human blood cDC1 and pDC after 42 h of culture. Cell sorter-purified cDC1, cDC2, CD14^-^ DC3, and pDC were incubated either with 400 ng/ml 8-MOP or equal amount of solvent control (ethanol) for 30 min at 37°C as indicated below the figure. Then, cells were either irradiated with 2 J/cm^2^ UV-A light or mock-treated. After centrifugation to remove the solvent, cells were resuspended in **(A)** medium or **(B)** medium containing 5 µg/ml R848. After 42 h of culture, DCs were stained with the antibodies used for cell sorting and 7-AAD and Annexin V-PE to determine viability. Truncated violin plots depict percentages of alive (Annexin V^-^/7-AAD^-^), early apoptotic (Annexin V^+^/7-AAD^-^), late apoptotic (Annexin V^+^/7-AAD^+^), and necrotic (Annexin V^-^/7-AAD^+^) cDC1 (yellow-orange symbols), cDC2 (red symbols), DC3 (purple symbols) and pDC (blue symbols) of five donors (each donor with an individual symbol). Statistical analysis was performed in GraphPad Prism (V10) using 2way ANOVA for grouped data with Dunnett’s multiple comparisons tests as posthoc test (*p < 0.05, **p < 0.01, ***p < 0.001, ****p < 0.0001).

**Figure 6:**
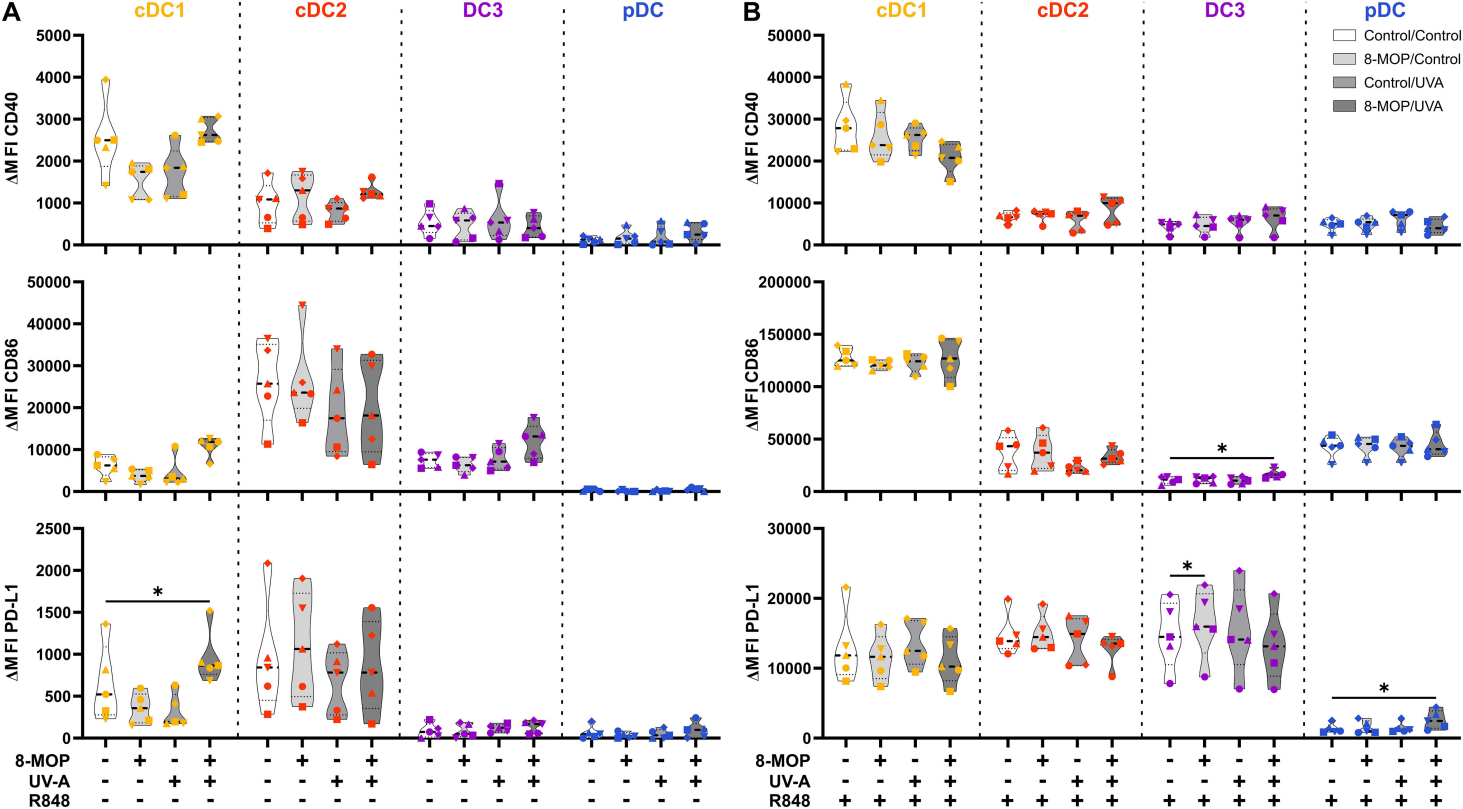
Experimental ECP induces minor changes in the expression of co-stimulatory and -regulatory molecules on human primary DCs after 42 h of culture. Cell sorter-purified cDC1, cDC2, CD14^-^ DC3, and pDC were incubated either with 400 ng/ml 8-MOP or equal amount of solvent control (ethanol) for 30 min at 37°C as indicated below the figure. Then, cells were either irradiated with 2 J/cm^2^ UV-A light or mock-treated. After centrifugation to remove the solvent, cells were resuspended in **(A)** medium or **(B)** medium containing 5 µg/ml R848. After 42 h of culture, DCs were stained with the antibodies used for cell sorting and A700-coupled anti-CD40, FITC-coupled anti-CD86, and BV650-coupled anti-PD-L1 or respective isotype controls. Truncated violin plots show ΔMFI on alive (Annexin V^-^/7-AAD^-^) cDC1 (yellow-orange symbols), cDC2 (red symbols), DC3 (purple symbols) and pDC (blue symbols) of five donors (each donor with an individual symbol). Statistical analysis was performed in GraphPad Prism (V10) using 2way ANOVA for grouped data with Dunnett’s multiple comparisons tests as posthoc test (*p < 0.05, **p < 0.01, ***p < 0.001, ****p < 0.0001).

In order to monitor how ECP would affect DCs at a later time point after reinfusion of the photophoresate, we performed the same analyses after 42 h of incubation at 37°C. The majority of DCs were apoptotic or necrotic in steady state conditions, but ECP reduced further the survival of CD14^-^ DC3 and pDC (**Figure 5A**). The low viability of the different DC subpopulations is in accordance with data on the circulating lifespan of human DCs *in vivo* showing a shorter lifetime of cDC1 (1.3 days) compared to DC2 and DC3 (2.2 days) (52). However, after TLR stimulation the survival of cDC1 (R848 and pIC) and pDCs (R848 and CpG) was strongly increased in control conditions, which was completely abrogated when cDC1 and pDC were treated with ECP *in vitro* (**Figure 5B**, **Supplementary Figure 4A**). In contrast, we did not observe enhanced cell death in cDC2 and CD14^-^ DC3 after *in vitro* ECP after stimulation with R848 (**Figure 5B**). As R848 can activate the NLRP3 inflammasome in human CD14^-^ DC3 (31), we used the TLR4 ligand CRX-527 to avoid the induction of cell death by the TLR ligand alone. Notably, *in vitro* ECP strongly induced cell death in CRX-527-activated CD14^-^ DC3 and to a minor extend in cDC2 (**Supplementary Figure 4B**). Thus, *in vitro* ECP seems to reduce the viability of cDC1 and pDC in general in response to TLR ligands, whereas it depends on the stimulated pattern recognition receptor for human cDC2 and CD14^-^ DC3. We also measured co-stimulatory and -inhibitory molecule expression by flow cytometry but did not observe strong changes on ECP-treated DCs compared to the control treatment (**Figure 6**). cDC1 showed enhanced expression of immunoregulatory PD-L1 in steady state conditions, whereas PD-L1 was enhanced on pDC after *in vitro* ECP upon stimulation irrespective of the TLR ligand (**Figure 6**, **Supplementary Figure 5B**). CD14^-^ DC3 showed slightly increased expression of the co-stimulatory molecule CD86 after R848 stimulation, when they were treated prior with ECP (**Figure 6**). The analysis of cytokine secretion showed that anti-inflammatory IL-1RA released by cDC2 and CD14^-^ DC3 and IL-10 released by cDC2 were increased by ECP upon TLR stimulation (**Figure 4B**). Overall, changes on phenotype and cytokine secretion by DCs due to ECP were low. Thus, *in vitro* ECP of human DC subpopulations showed minor effects on the phenotype of the DCs but strongly induced apoptosis in cDC1 and pDCs after activation by TLR ligands as well as in CD14^-^ DC3 dependent on the used TLR ligand.

### 3.2 ECP reduces the capacity of human blood cDC1 to activate memory T cell responses

**Figure 7:**
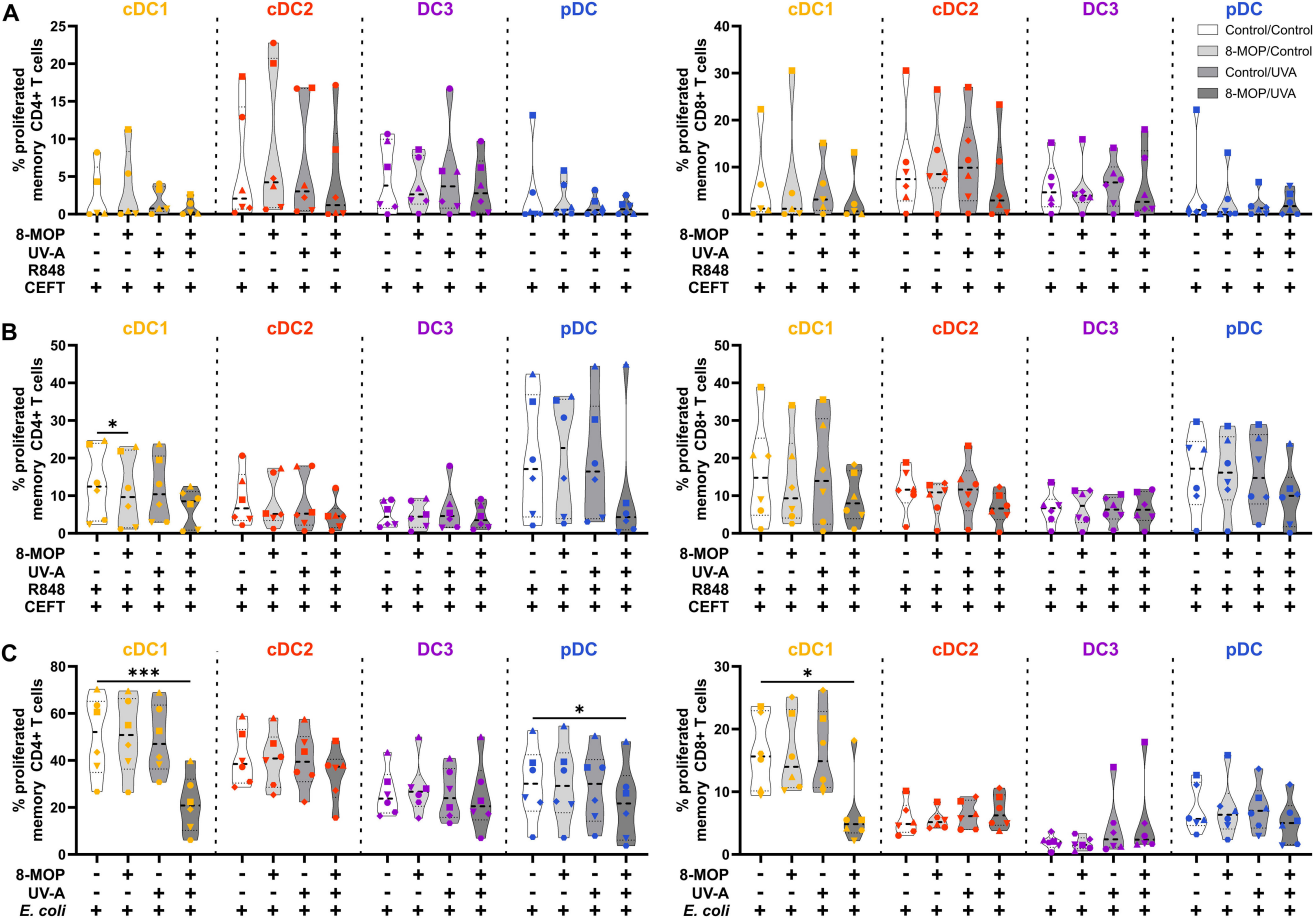
*In vitro* ECP of human primary blood cDC1 strongly reduces their capacity to activate memory T cells. Cell-sorted cDC1 (yellow-orange), cDC2 (red), CD14^-^ DC3 (purple), and pDC (blue) were treated with 8-MOP and UV-A light. After washing, DCs were **(A)** pulsed with CEFT peptides, **(B)** pulsed with CEFT peptides in presence of 1 µg/ml R848, or **(C)** incubated with 10 CFU/DC heat-killed *E. coli*. After 18 h of culture, autologous CFSE-labelled memory T cells were added (1:10 DC:T cell ratio) and co-cultured for five days. T cells were stained with a panel of fluorochrome-coupled antibodies and acquired using a Cyoflex S (Beckman Coulter). T cells were gated as shown in **Supplementary Figure 6**. Truncated violin plots depict percentages of proliferated and activated (CFSE^-^CD25^+^) CD4^+^ (left panel) and CD8^+^ (right panel) memory T cells of six donors (cDC1 in **(A)** five donors; each donor with an individual symbol). Statistical analysis was performed in GraphPad Prism (V10) using 2way ANOVA for grouped data with Dunnett’s multiple comparisons tests as posthoc test (*p < 0.05, **p < 0.01, ***p < 0.001, ****p < 0.0001).

**Figure 8:**
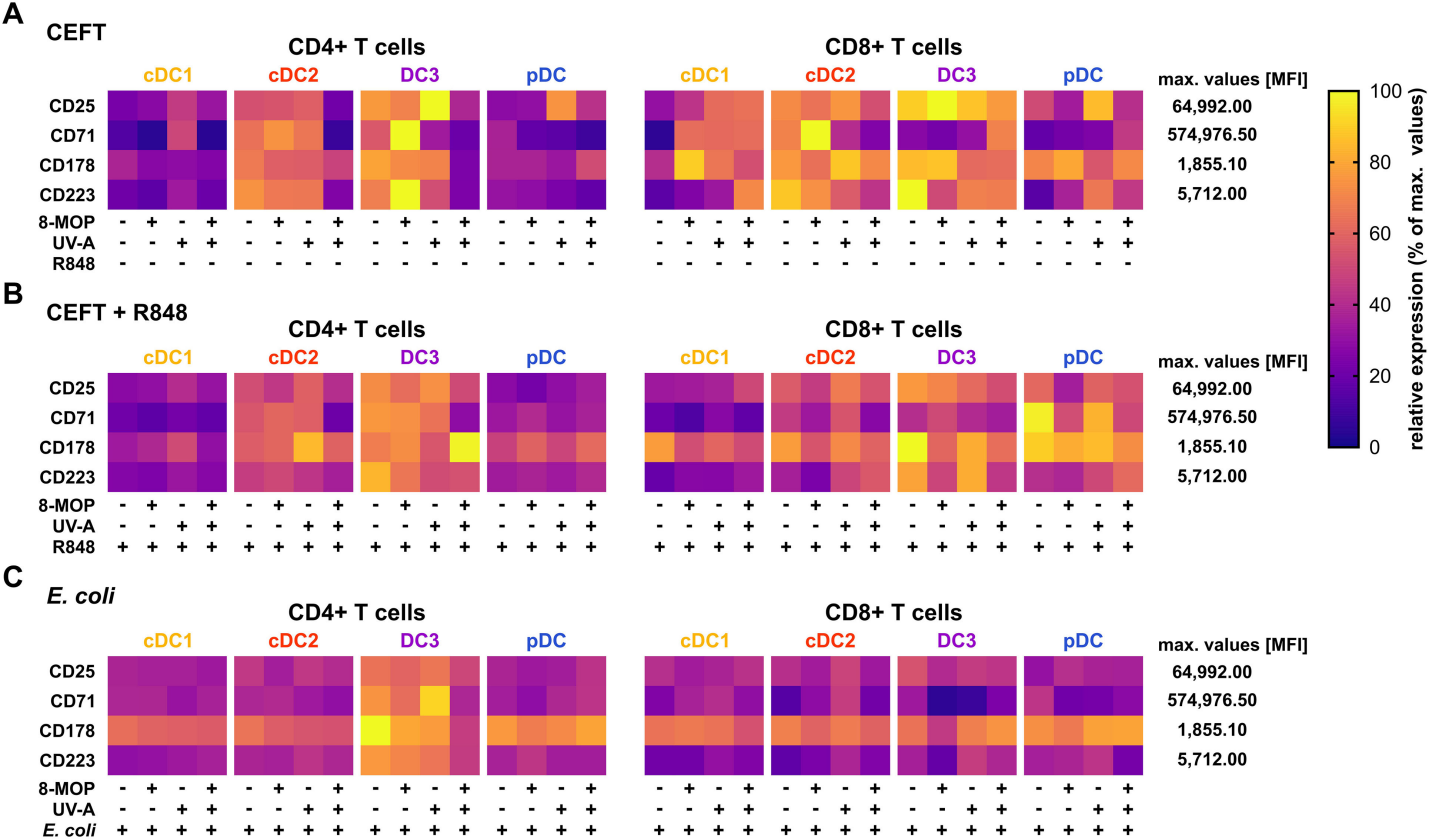
Experimental ECP of primary blood DCs does not enhance the expression of exhaustion markers or death receptors on activated memory T cells. Proliferated and activated memory T cells from **Figure 7** were analyzed for the expression of CD25, CD71, CD178, and CD223 by flow cytometry. Data were normalized to the highest measured value in the data set and the relative values (percentage of maximum) plotted as heatmap. Each square shows the mean of six donors (cDC1 in **(A)** five donors).

**Figure 9:**
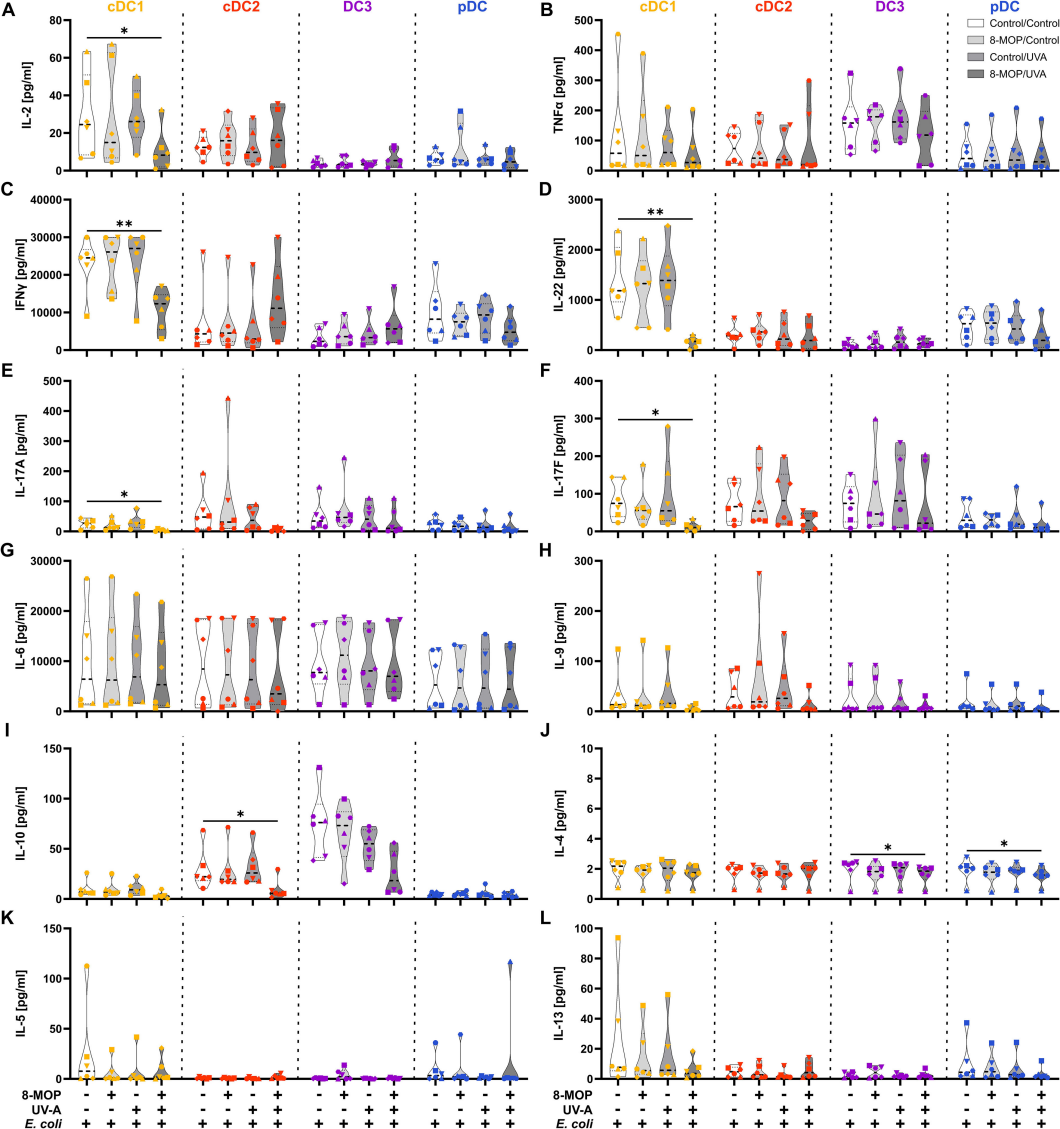
*In vitro* ECP-treatment of human cDC1 reduces the secretion of TH1- and TH17-associated cytokines by memory T cells. Supernatants from DC:T cell co-cultures shown in **Figure 9C** were analyzed for the concentration of T cell-associated cytokines using the LEGENDplex Hu Th Cytokine Panel (BioLegend). Truncated violin plots show the concentrations of **(A)** IL-2, **(B)** TNFα, **(C)** IFNγ, **(D)** IL-22, **(E)** IL-17A, **(F)** IL-17F, **(G)** IL-6, **(H)** IL-9, **(I)** IL-10, **(J)** IL-4, **(K)** IL-5, and **(L)** IL-13 for T cells co-cultured with cDC1 (yellow-orange symbols), cDC2 (red symbols), DC3 (purple symbols) and pDC (blue symbols) of six donors (each donor with an individual symbol). Statistical analysis was performed in GraphPad Prism (V10) using 2way ANOVA for grouped data with Dunnett’s multiple comparisons tests as posthoc test (*p < 0.05, **p < 0.01, ***p < 0.001, ****p < 0.0001).

Since it has been reported that ECP induces amelioration of different T cell-mediated diseases, such as GvHD, we were interested in whether *in vitro* ECP would influence the T cell stimulatory capacity of human DCs. As ECP affects preexisting immune responses in patients, we analyzed the T cell stimulatory capacity of DCs by co-culturing them with autologous CD4^+^ and CD8^+^ memory T cells in the presence of common viral and bacterial antigens. DCs were sorted after negative enrichment and memory T cells were purified from PBMCs of the same donor by magnetic bead-based enrichment kits. Then, sorted DCs were treated with *in vitro* ECP as described above. They were either loaded with a pool of pre-processed MHCI- and MHCII-specific peptides derived from common antigens (CEFT: CMV, EBV, Influenza, and Tetanus Toxoid) which can be loaded on a broad array of HLA molecules with or without additional stimulation with the TLR ligand R848 as it strongly activates all human DC subpopulations. As loading with peptides is a passive process that does not require processing of antigens, we were interested in how *in vitro* ECP would influence the capacity to process antigens. Here, the sorted DCs were incubated with heat-killed *E. coli* that have to be phagocytosed and processed in order to present peptides on HLA molecules to T cells. Further, as *E. coli* is a complex pathogen, it is able to activate diverse pattern recognition receptors, such as TLR2, TLR4, and TLR8 (53–57). Thereby, influence of the activated TLR on cell death induction might be minimized. After 18 h of culture with the peptides or *E. coli*, CFSE-labelled memory T cells were added and co-cultured with the sorted DCs for five days. Then, T cells were analyzed by flow cytometry for proliferation (dilution of CFSE signal), activation (expression of CD25 and CD71), and phenotype (expression of CD178, CD223, and CCR7) (**Supplementary Figure 6**). When DCs were loaded with CEFT peptides in steady state conditions, the DCs could hardly activate memory CD4^+^ and CD8^+^ T cells and we did not observe any influence of *in vitro* ECP on the induction of memory T cell proliferation (**Figure 7A**). Simultaneous stimulation and peptide loading (CEFT + R848) boosted the capacity of cDC1 and pDC to induce memory T cell proliferation but *in vitro* ECP showed only a minor reduction by cDC1 and pDC to activate memory T cells (**Figure 7B**). However, when DCs were cultured with heat-killed *E. coli*, which have to be phagocytosed and processed in order to restimulate *E. coli*-reactive memory T cells, we observed a strong decline in proliferated memory CD4^+^ and CD8^+^ T cells when cDC1 were exposed to *in vitro* ECP (**Figure 7C**). ECP also reduced the capacity of pDC to activate memory CD4^+^ T cells (**Figure 7C**). While *in vitro* ECP did not influence the capacity of cDC2 and CD14^-^ DC3 to induce T cell proliferation (**Figure 7C**), we observed changes of the phenotype of the proliferated CD4^+^ T cells (**Figure 8**). T cells stimulated by steady state cDC2 and CD14^-^ DC3 showed lower expression of the activation markers CD25 and CD71 as well as of the exhaustion marker CD223 (LAG-3), when DCs were treated with ECP prior to the co-culture (**Figure 8A**). This was also the case when CD14^-^ DC3 were stimulated with heat-killed *E. coli* prior to the co-culture with memory T cells (**Figure 8C**). As ECP modulates the polarization of T cells (8, 10), we were interested whether ECP-treated DCs influence the secretion of cytokines by activated T cells. Therefore, we analyzed the supernatants of DC:T cell co-culture for cytokines associated with different subsets of T helper cells by CBA assay. When DCs were only loaded with CEFT peptide without TLR stimulation, levels of secreted cytokines were low and not influenced by ECP-treatment (**Supplementary Figure 7**). When DCs were simultaneously activated during peptide loading, we observed higher level of TH1-associated cytokines such as IFNγ but they were not influenced by the treatment with ECP (**Supplementary Figure 8**). However, in accordance with the T cell proliferation data (**Figure 7C**), we observed a strong reduction in TH1- and TH17-associated cytokines (IFNγ, IL-22, IL-17A, and IL-17F) as well as IL-2, when cDC1 were stimulated with *E. coli* after pretreatment with *in vitro* ECP (**Figure 9**). In contrast, *in vitro* ECP did not influence the secretion of cytokines when T cells were co-cultured with cDC2, CD14^-^ DC3 and only slightly with pDC (**Figure 9**). Further, *in vitro* ECP did not influence the secretion of TH2- (IL-4, IL-5, IL-13) or Treg-associated cytokines (IL-10) irrespective of the DC subset and the antigen (**Figure 9**, **Supplementary Figures 7**, **8**). Thus, *in vitro* ECP of human primary blood DCs directly influences the capacity of cDC1 and pDC to induce memory T cell activation as well as changes the phenotype of memory T cells activated by cDC2 and CD14^-^ DC3. Further, it reduces the secretion of TH1- and TH17-associated cytokines by T cells, when restimulated with ECP-treated cDC1.

## 4 Discussion

ECP is a widely used immunomodulating therapy for various T cell-mediated diseases, such as CTCL, GvHD, and allograft rejection. Although ECP has been used for more than 30 years, the exact mechanism of action remains enigmatic. While it has been known that ECP induces apoptosis in treated leukocytes, analysis of the response of human primary DCs to ECP was hampered by the scarcity of the cells in human blood. In this study, we show that ECP induces apoptosis in the DC subpopulations cDC1 and pDC upon TLR stimulation, whereas cDC2 and CD14^-^ DC3 are less affected. While ECP has only a minor influence on the expression of co-stimulatory and -regulatory molecules as well as on the secretion of cytokines by DCs, it strongly reduces the capacity of cDC1 and - to a minor extent - of pDC to activate memory T cell responses. Further, secretion of TH1- and TH17-associated cytokines after co-culture with ECP-treated cDC1 were strongly diminished. Since especially cDC1 have a pivotal role in the T cell-mediated immune response, our data suggest that reducing the function of cDC1 might contribute to the immunomodulating effect of ECP.

Currently, DCs in mice and men are classified in four main DC subpopulations, namely cDC1, cDC2, DC3, and pDC, with functional specialization. In our experimental ECP model, we observed the strongest effect on human cDC1 with apoptosis induction as well as reduced activation of CD4^+^ and CD8^+^ memory T cell responses. cDC1 are known for their high capacity to cross-present antigens to CD8^+^ T cells as well as the ability to prime TH1 cells (17, 18, 58–61). Therefore, they are crucial for the induction of anti-tumor T cell responses (14, 20, 21). However, the role of cDC1, and DCs in general, is controversial in GvHD (62–69). Host and donor DCs seem to be necessary for the induction of GvHD and additional transfusion of DCs deteriorates the symptoms of GvHD (62, 68, 69). Further, the depletion of DCs ameliorates the disease indicating a GvHD-promoting effect of DCs (63, 64). Based on these studies, induction of apoptosis and reducing the T cell-stimulatory capacity of DCs might contribute to the positive effects of ECP in the treatment of GvHD. However, other studies demonstrated rather protective effects of cDC1 and pDCs against GvHD (65–67, 70). In mice, expansion of cDC1 by FLT3L injection prior to bone marrow (BM) transfer reduced GvHD mortality by clonal deletion of alloreactive T cells (66, 67). Moreover, transfer of tolerogenic CCR9^+^ pDCs together with the BM reduced GvHD mortality by inducing Tregs (70). Batf3^-/-^ mice lacking selectively the cDC1 population showed aggravated GvHD and faster mortality compared to wild type mice (65). Thus, the role of DCs in murine models of GvHD is still unclear. However, our results are in accordance with data from a murine model of contact hypersensitivity (45). Here, the transfer of ECP-treated enriched DCs was sufficient to suppress antigen-specific T cell responses (45). While several studies showed enhanced induction of Tregs after therapy with ECP (10, 43, 71–73), we observed mainly a decrease in TH1- and TH17-associated cytokines by *in vitro* ECP of human cDC1. As we restimulated the already polarized memory T cells only once with ECP-treated DCs, this might not be sufficient for a repolarization of TH1 or Th17 cells into Tregs as observed in patients treated with ECP over a longer period of time.

In contrast to T cell-mediated inflammatory diseases such as GvHD, treatment of CTCL patients with ECP rather induces T cell responses against the lymphoma cells by differentiation of monocytes into DC-like cells (50, 74, 75). However, the differentiation of monocytes to DC-like cells is not directly dependent on treatment with 8-MOP and UV-A light but rather on the interaction of monocytes with platelets in the device which are activated due to the plastic surface (49). While DCs in GvHD patients show rather tolerogenic responses to ECP (42, 43, 51), the monocyte-derived DC-like cells in CTCL patients are rather proinflammatory and thought to induce anti-lymphoma T cell responses (50, 74, 76). Whether these controversial reports are due to cell type-specific responses to ECP (monocytes vs. DCs) or because of different environments in patients (suppressive TH2 prone environment in CTCL vs. inflammatory TH1 prone environment in GvHD) is not clear yet. However, we also observed subtype-specific reaction to *in vitro* ECP, as monocyte-related DC3 showed a more proinflammatory phenotype, whereas bona fide cDC1 underwent apoptosis and lost their T cell stimulatory capacity (77). These differences might also explain why treatment of GvHD patients with ECP is not associated with an increased risk for infections and has less severe side effects than immunosuppressive regimens (78, 79). As cDC2 and CD14^-^ DC3 still have the capacity to activate T cells, they might be responsible for the induction of immune responses against invading pathogens and compensate for the loss of cDC1.

In order to analysis the impact of the underlying disease on the response of the primary DCs to ECP, DCs have to be isolated from patients suffering from CTCL or GvHD. However, this is currently not possible due to the scarcity of DCs. We additionally observed an influence of ECP on pDCs. Due to our gating strategy (see **Figure 1**), we cannot exclude that the sorted pDC contain transitional DCs (tDC). tDC have a pDC-like phenotype based on marker expression such as CD123 and CD303 (BDCA-2) but have the potential to differentiate into DC2-like cells (80–84). However, we did not observe the emergence of CD1c^+^ cells during the culture of sorted pDC (**Supplementary Figure 1**) implying that either tDC were depleted during the enrichment process or the time frame of the experiments was too short for efficient differentiation of tDC into DC2-like cells. To exclude contamination with tDC in future studies, Axl might be added to the staining panel used for cell-sorting.

While we concentrated in our study on the direct influence of ECP on DC subpopulations, it is clear that ECP in certain conditions has an indirect effect on the immune system via the recognition of apoptotic lymphocytes by immune cells. When ECP is used to treat immune-related adverse events in cancer patients due to treatment with immune checkpoint inhibitors, the apoptotic leukocytes induced by ECP are ingested by intestinal macrophages leading to an anti-inflammatory M2-like polarization of macrophages by STAT6 signaling (7). This induces the secretion of adiponectin and the subsequent expression of arginase-1 leading to tolerogenic T cell responses (7). ECP-treated apoptotic lymphocytes induced a tolerogenic phenotype in untreated BMDCs and moDCs in an *in vitro* model, thereby leading to increased Treg induction in a cardiac allograft rejection model and a mixed lymphocyte response, respectively (43, 71). Thus, ECP might additionally induce a tolerogenic phenotype in untreated DCs by the recognition of apoptotic cells, which might be analyzed in future studies. However, this is only possible in cultures of whole PBMCs that are treated with ECP. Unambiguous identification of DC subpopulations in cultures of whole PBMCs is not possible over a longer period of time, as the surface marker expression on DCs as well as on monocytes changes during the culture and due to the scarcity of DCs. Therefore, we were limited to analyze effects of ECP on DCs in cultures of cell-sorter purified DCs of healthy donors. Thus, we cannot exclude that the presence of other cells and the secretion of soluble molecules by ECP-treated leukocytes might influence the response of DCs to ECP. In order to mimic inflammatory conditions present in GvHD, we used the TLR ligand R848 in our study. However, the environment in GvHD patients is very complex and additionally influenced by the treatment with corticosteroids and other immunosuppressive drugs. Nevertheless, TLR signaling is involved in GvHD pathogenesis with contributions of different TLRs, such as TLR4, TLR7, and TLR9 (85–91). In conclusion, we demonstrate that ECP directly induced apoptosis in cDC1 and pDC upon TLR stimulation and strongly reduced the capacity of especially cDC1 to activate memory CD4^+^ and CD8^+^ T cell responses.

## Data Availability

The raw data supporting the conclusions of this article will be made available by the authors, without undue reservation.
