# Supplementary material for: Extracorporeal photopheresis reduces the T cell stimulatory capacity of human primary blood conventional dendritic cells type 1
Source: Front Immunol. 2025 Aug 13;16:1646421. doi: 10.3389/fimmu.2025.1646421 (PMC12380567; doi:10.3389/fimmu.2025.1646421)
Supplement: Supplementary file 1 [file DataSheet1.pdf]

frontiers

Supplementary Material

Extracorporeal photopheresis reduces the T cell stimulatory capacity of human primary blood conventional dendritic cells type 1

Lukas Heger[1*], Carola Berking[2,3], Holger Hackstein[1]

[1]Department of Transfusion Medicine and Hemostaseology, Universitätsklinikum Erlangen, Friedrich-Alexander-Universität Erlangen-Nürnberg, Erlangen, Germany

[2]Department of Dermatology, Universitätsklinikum Erlangen, Friedrich-Alexander-Universität Erlangen-Nürnberg, Erlangen, Germany

[3]Deutsches Zentrum Immuntherapie, Universitätsklinikum Erlangen, Friedrich-Alexander-Universität Erlangen-Nürnberg, Erlangen, Germany

*** Correspondence:**
Dr. Lukas Heger
Lukas.heger@uk-erlangen.de

1 Supplementary Figures

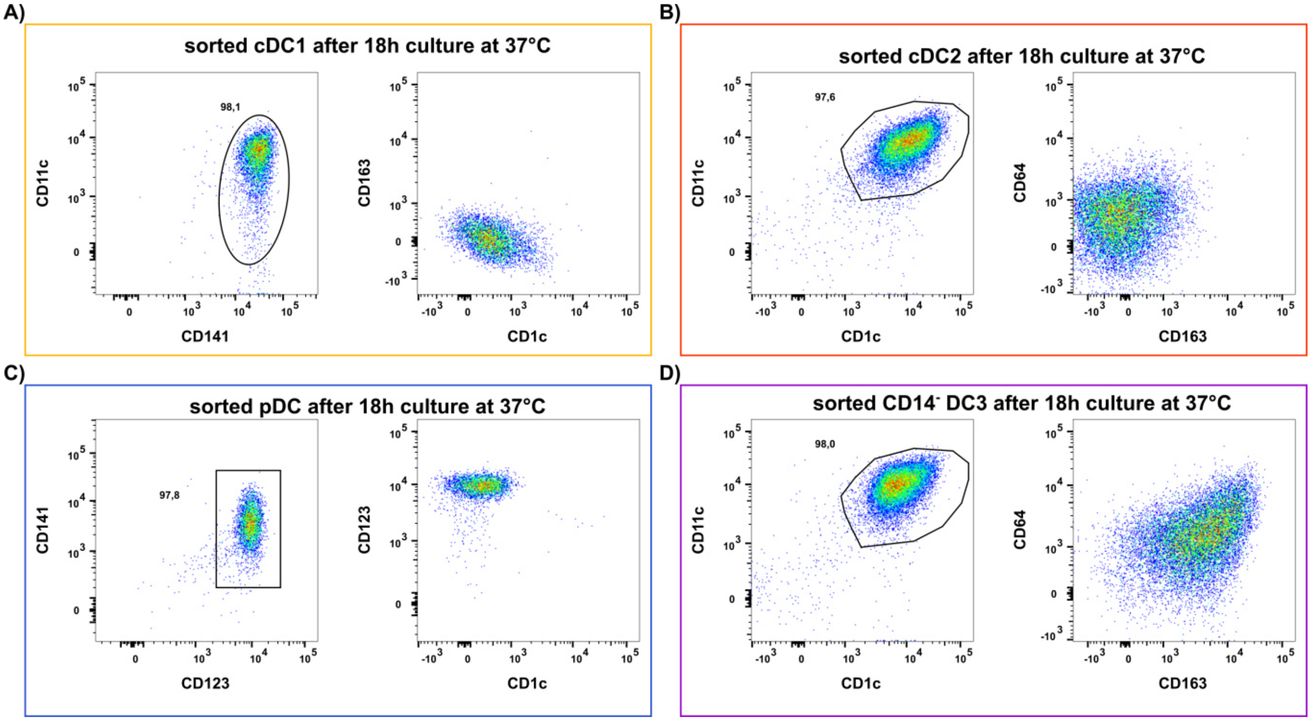

Supplementary Figure 1: Phenotype of sorted DCs after 18h of culture. cDC1, cDC2, $CD14^-$ DC3, and pDC were sorted from blood of healthy donors and treated with 8-MOP or solvent control (ethanol) followed by UV-A light irradiation or Mock-treatment. After 18h, cells were stained with fluorochrome-coupled antibodies used for cell-sorting and analyzed using a Cytoflex S (Beckman Coulter). After gating for morphology of dendritic cells (FSC-A/SSC-A) and single cells (FSC-A/FSC-H), APCs ($HLA\text{-}DR^+$) were selected and gated for Annexin V and 7-AAD as shown in Supplementary Figure 2. Viable cells (Annexin V^-7-AAD^-) were gated. Pseudocolor dotplots show the gating for the respective cell population (left panel) and the phenotype of the cells for other DC subset defining markers (right panel) for (A) cDC1, (B) cDC2, (C) pDC, and (D) $CD14^-$ DC3.

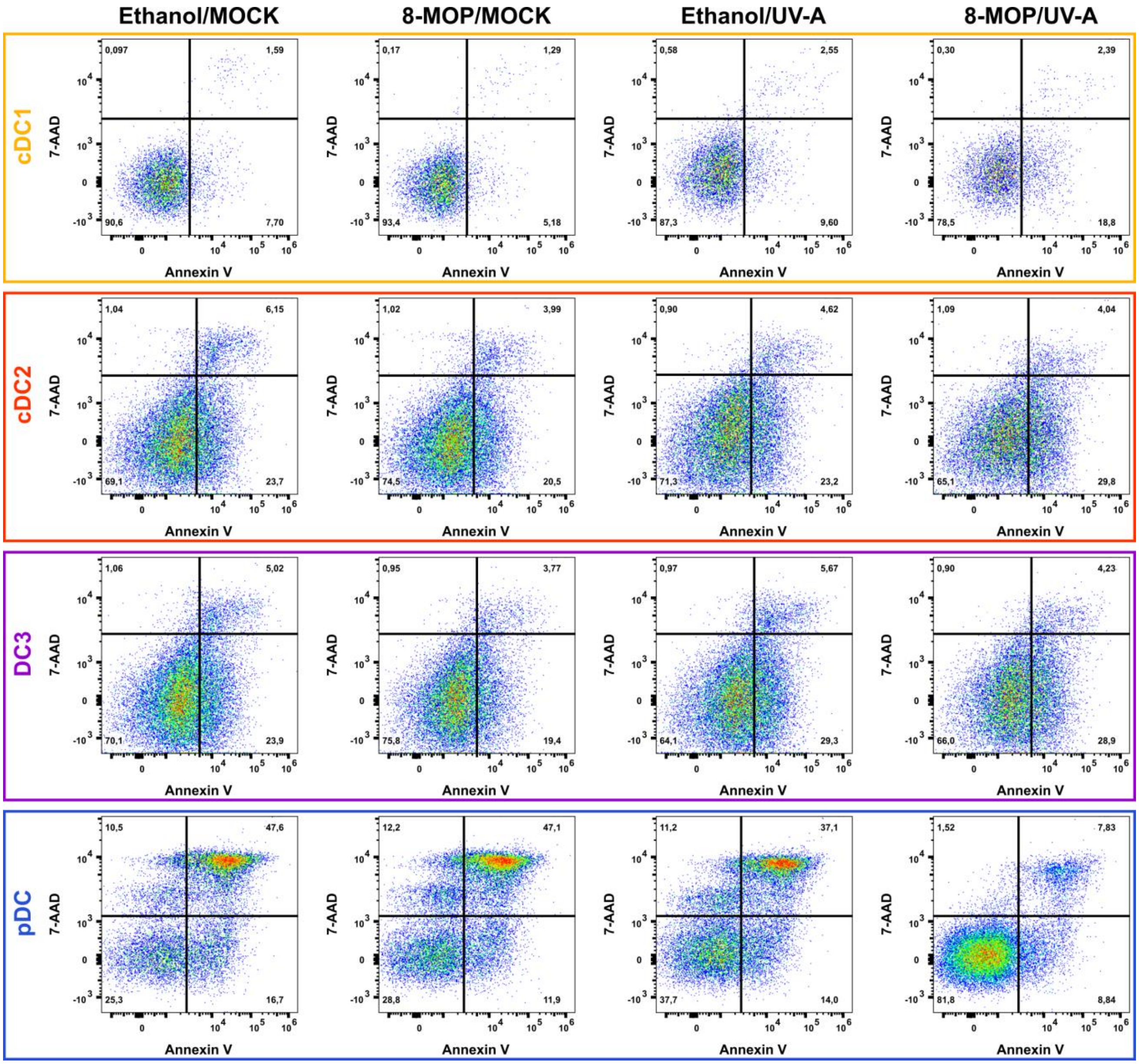

Supplementary Figure 2: Identification of cell death in sorted DCs by Annexin V/7-AAD staining. cDC1, cDC2, CD14$^-$ DC3, and pDC were sorted from blood of healthy donors and treated with 8-MOP or solvent control (ethanol) followed by UV-A light irradiation or Mock-treatment. After 18h, cells were stained with Annexin V and 7-AAD to identify cell death. Shown are pseudocolor dot plots of sorted cDC1 (upper panel), cDC2 (second panel), DC3 (third panel), and pDC (lowest panel). Based on quadrant gating, DCs were assigned either to early apoptotic (Annexin V$^+$7-AAD$^-$), late apoptotic (Annexin V$^+$7-AAD$^+$), necrotic (Annexin V$^-$7-AAD$^+$), or viable cells (Annexin V$^-$7-AAD$^-$). Frequency of parent was determined for each quadrant using the statistics function of FlowJo.

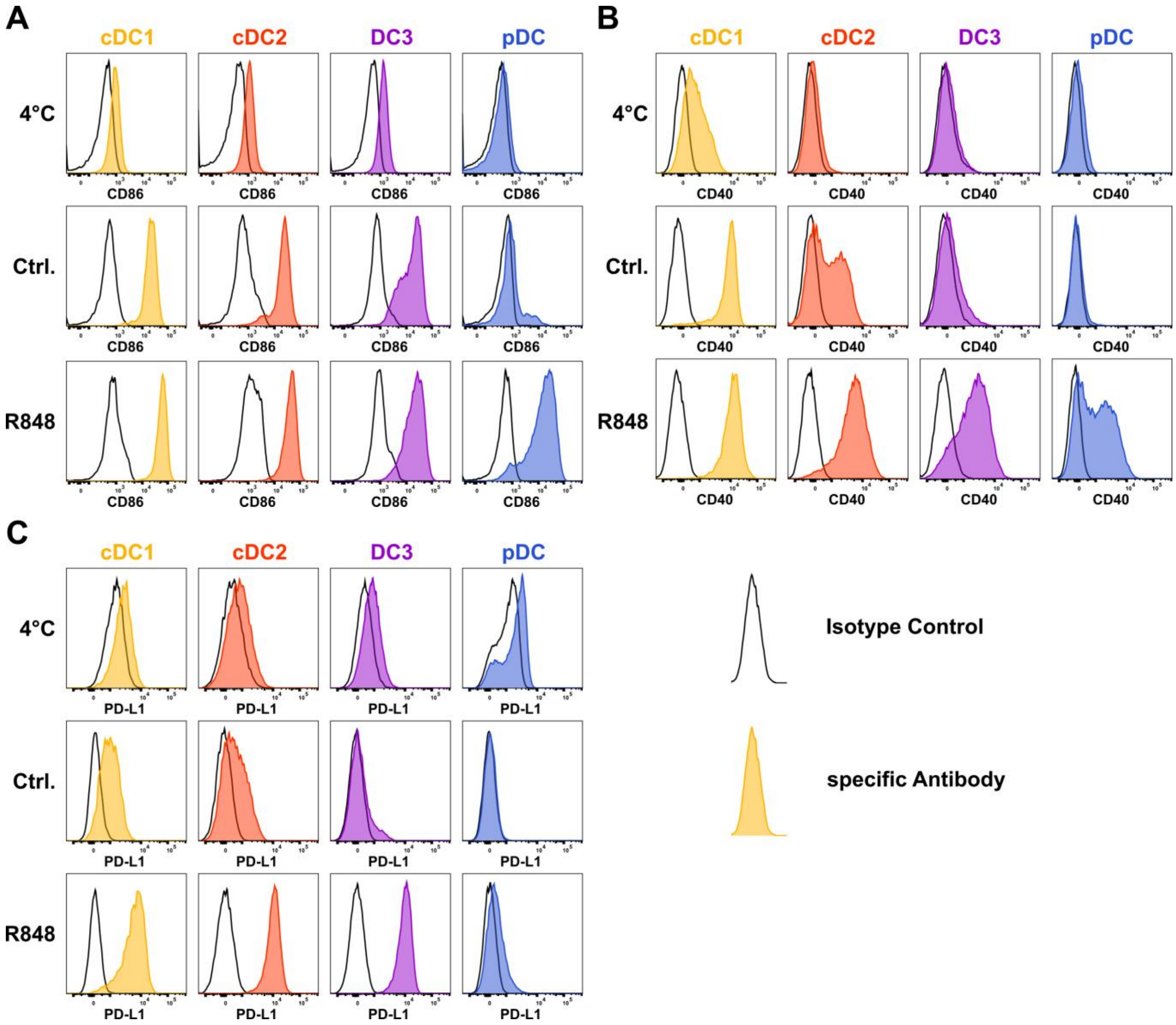

Supplementary Figure 3: Representative overlay histograms for the expression of co-stimulatory molecules on human DCs. Human cDC1, cDC2, CD14$^-$ DC3, and pDC were cell-sorted from blood of healthy donors and treated with 8-MOP or solvent control (ethanol) followed by UV-A light irradiation or Mock-treatment. Then, cells were stimulated (lower panel) or not (middle panel) with 5 µg/ml R848. As control, cells were kept at 4°C until analysis (upper panel). After 18h, cells were stained with fluorochrome-coupled antibodies used for cell-sorting as well as antibodies against (A) CD86, (B) CD40, or (C) PD-L1 or respective isotype control. A-C) Overlay histograms show expression of co-stimulatory or -regulatory molecules (filled histograms, colored) in comparison to isotype controls (open histogram, black line) for cDC1 (yellow-orange), cDC2 (red), DC3 (purple), and pDC (blue) of one representative donor.

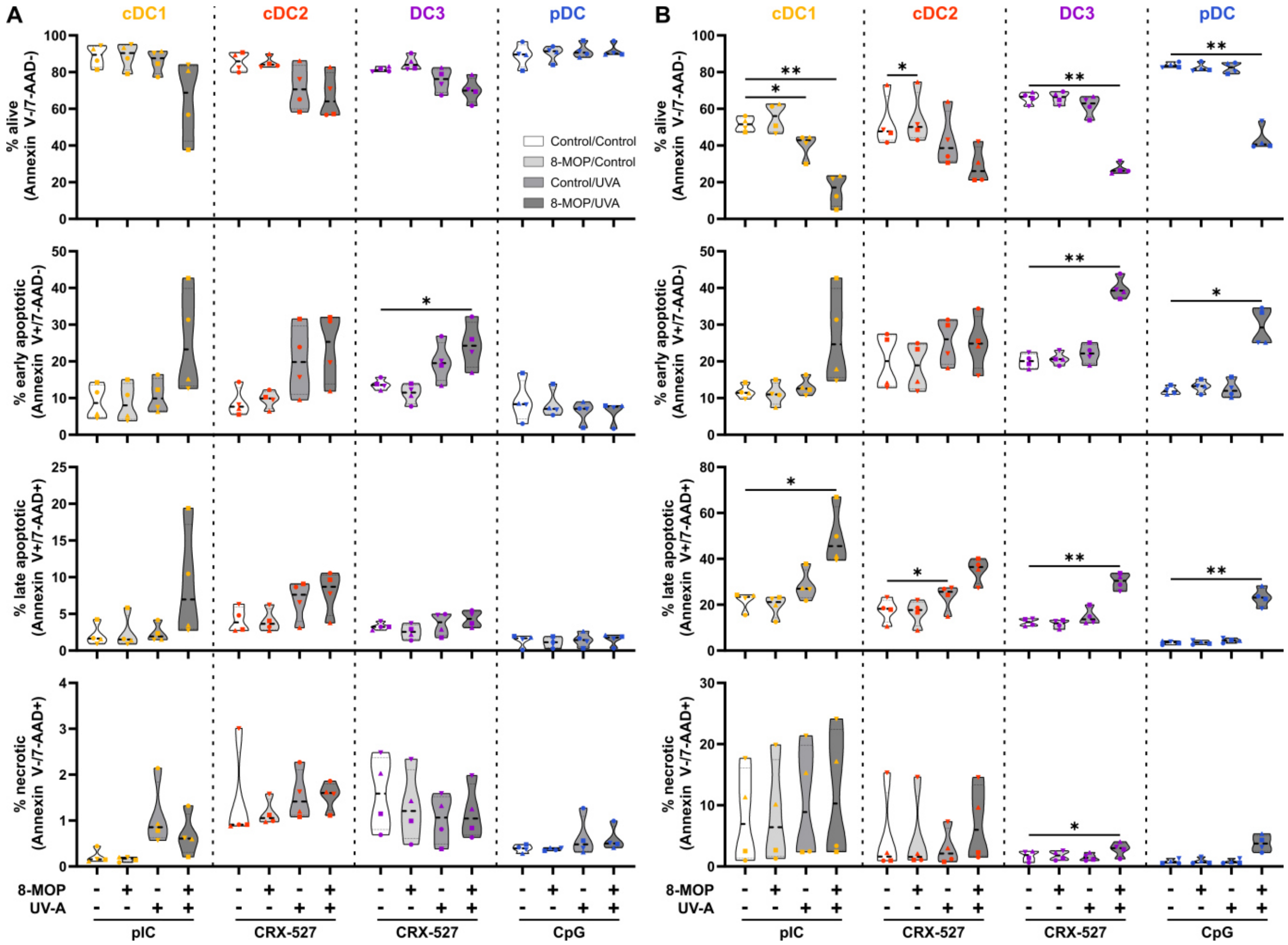

Supplementary Figure 4: TLR3-, TLR4-, and TLR9 ligands render human DC subpopulations sensitive to apoptosis induction following *in vitro* ECP. Cell sorter-purified cDC1, cDC2, CD14$^-$ DC3, and pDC were incubated either with 400 ng/ml 8-MOP or equal amount of solvent control (ethanol) for 30 min at 37°C as indicated below the figure. Then, cells were either irradiated with 2 J/cm^2 UV-A light or mock-treated. After centrifugation to remove the solvent, cells were resuspended in medium containing 5 µg/ml pIC (cDC1), 100 ng/ml CRX-527 (cDC2, CD14$^-$ DC3), or 2 µM CpG (ODN2216; pDC). After (A) 18 h or (B) 42 h of culture, DCs were stained with the antibodies used for cell sorting and 7-AAD and Annexin V-PE to determine viability. Truncated violin plots depict percentages of alive (Annexin V$^-$/7-AAD$^-$), early apoptotic (Annexin V$^+$/7-AAD$^-$), late apoptotic (Annexin V$^+$/7-AAD$^+$), and necrotic (Annexin V$^-$/7-AAD$^+$) cDC1 (yellow-orange symbols), cDC2 (red symbols), DC3 (purple symbols) and pDC (blue symbols) of four donors (each donor with an individual symbol). Statistical analysis was performed in GraphPad Prism (V10) using 2way ANOVA for grouped data with Dunnett's multiple comparisons tests as posthoc test (* $p < 0.05$, ** $p < 0.01$, *** $p < 0.001$, **** $p < 0.0001$).

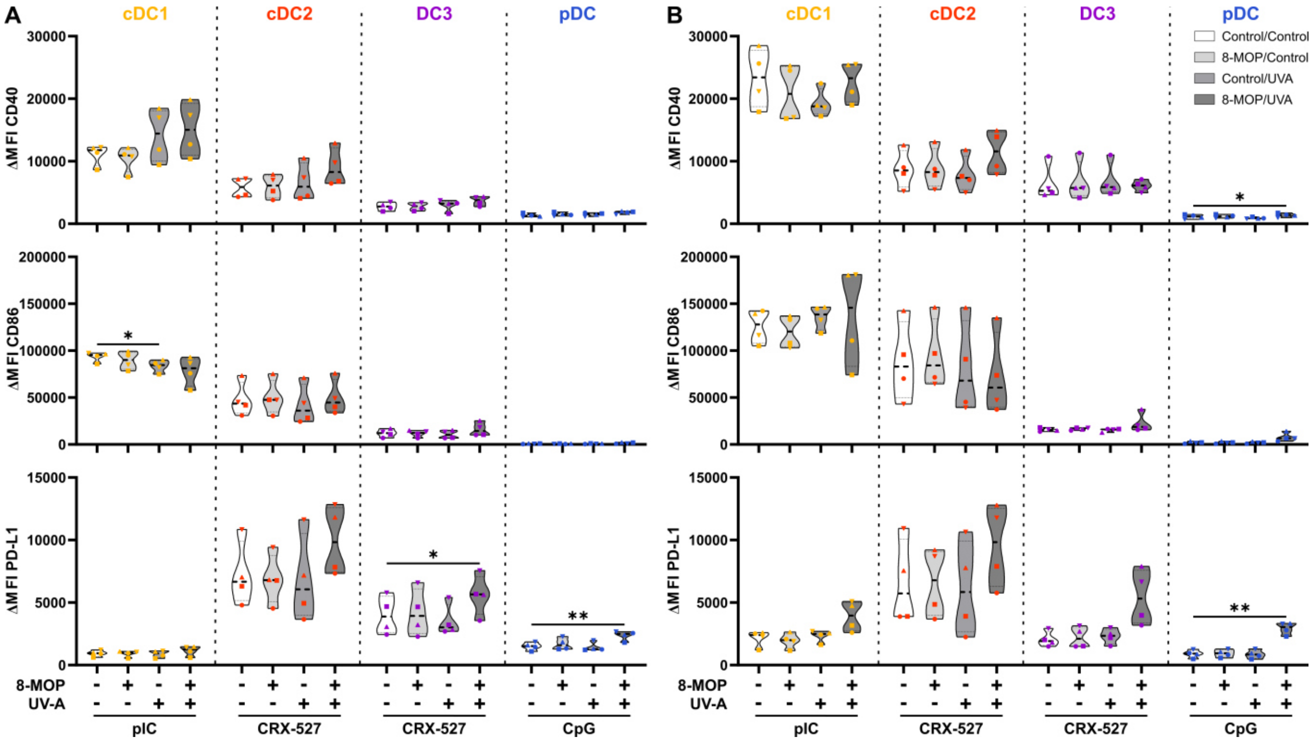

Supplementary Figure 5: *In vitro* ECP only slightly modulates co-stimulatory and -regulatory molecule expression on human DCs after stimulation of TLR3, TLR4, or TLR9. Cell sorter-purified cDC1, cDC2, $CD14^-$ DC3, and pDC were incubated either with 400 ng/ml 8-MOP or equal amount of solvent control (ethanol) for 30 min at 37°C as indicated below the figure. Then, cells were either irradiated with 2 J/cm^2 UV-A light or mock-treated. After centrifugation to remove the solvent, cells were resuspended in medium containing 5 µg/ml pIC (cDC1), 100 ng/ml CRX-527 (cDC2, $CD14^-$ DC3), or 2 µM CpG (ODN2216; pDC). After (A) 18 h or (B) 42 h of culture, DCs were stained with the antibodies used for cell sorting as well as antibodies against CD40, CD86, and PD-L1 or respective isotype contrls. Truncated violin plots show ΔMFI values for cDC1 (yellow-orange symbols), cDC2 (red symbols), DC3 (purple symbols) and pDC (blue symbols) of four donors (each donor with an individual symbol). Statistical analysis was performed in GraphPad Prism (V10) using 2way ANOVA for grouped data with Dunnett's multiple comparisons tests as posthoc test (* $p < 0.05$, ** $p < 0.01$, *** $p < 0.001$, **** $p < 0.0001$).

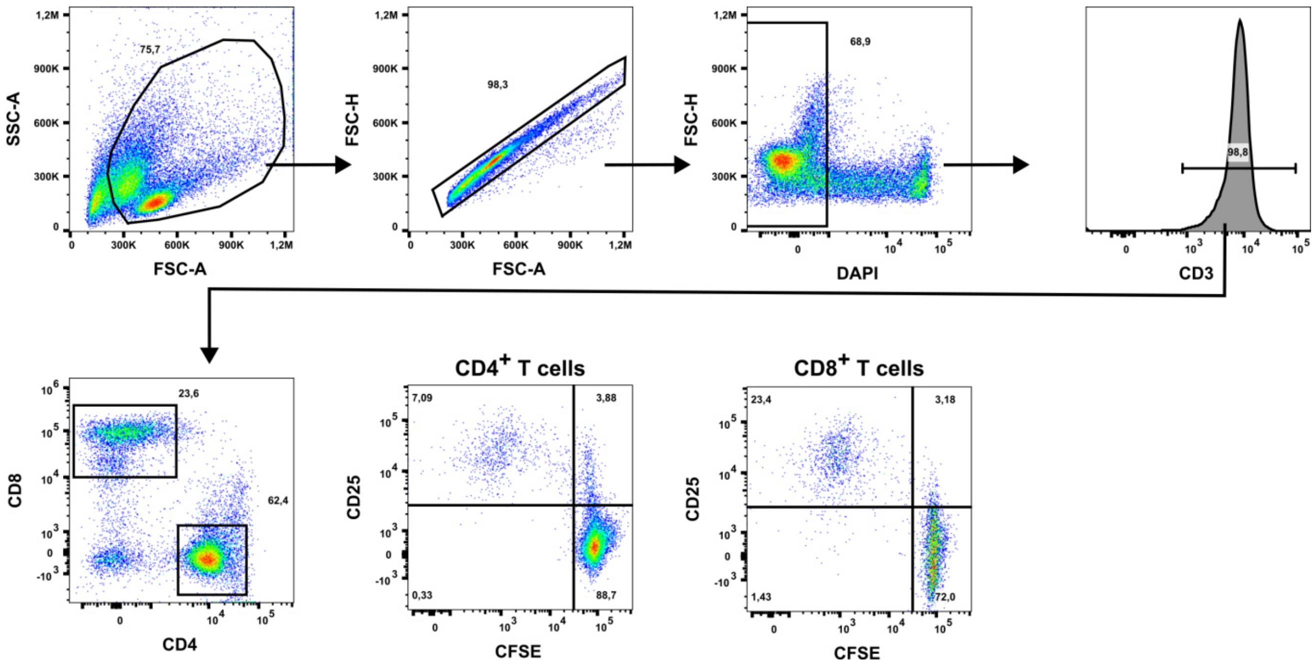

Supplementary Figure 6: Applied gating strategy for the identification of activated and proliferated CD4$^+$ and CD8$^+$ T cells. Enriched and CFSE-labelled CD4$^+$ and CD8$^+$ memory T cells were co-cultured with cell-sorted DCs after *in vitro* ECP treatment followed by loading with CEFT peptides with and without TLR stimulation or stimulation with heat-killed *E. coli* for 18 h. After five days of co-culture, T cells were stained with fluorochrome coupled antibodies as well as DAPI. Cells were acquired using a Cytoflex S (Beckman Coulter) and analyzed using FlowJo Software. For the analysis shown in Figure 7 and 8, cells were gated as shown: After a morphology gate for lymphocytes (FSC-A/SSC-A), doublets were excluded using FSC-A/FSC-H). Living (DAPI$^-$) were selected and T cells gated based on CD3 expression. Then, T cells were divided into CD4$^+$ and CD8$^+$ T cells and analyzed for proliferation (dilution of CFSE) and activation (expression of CD25) using a quadrant gate. Cells in the upper left quadrant (CFSE$^-$CD25$^+$) were shown in Figure 7 (frequency) and analyzed for the expression of surface markers shown in Figure 8.

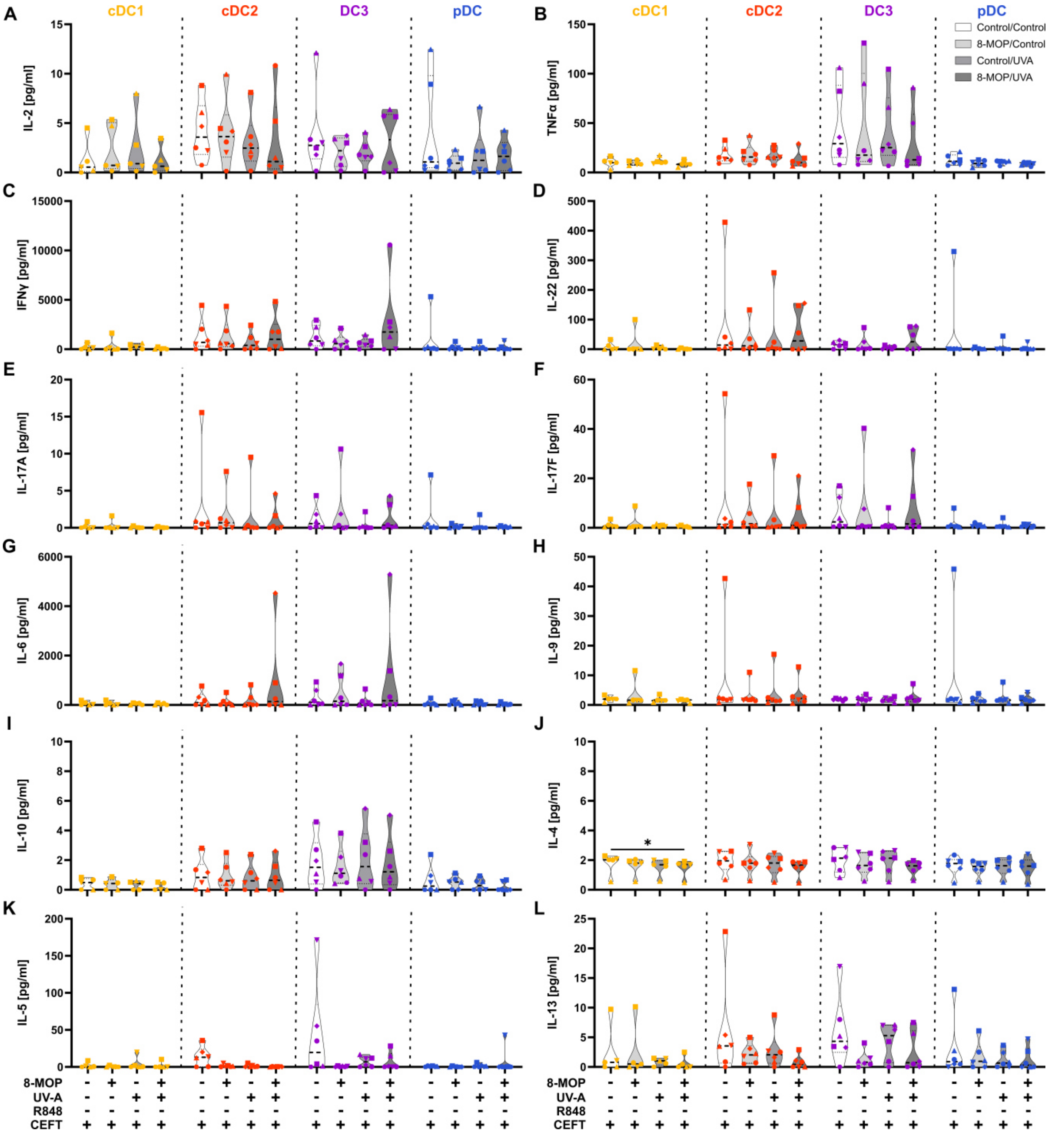

Supplementary Figure 7: Memory T cells restimulated by CEFT-loaded human DCs without TLR stimulation produce limited amounts of cytokines. Supernatants from DC:T cell co-cultures shown in Figure 7A were analyzed for the concentration of T cell-associated cytokines using the LEGENDplex Hu Th Cytokine Panel). Truncated violin plots show the concentrations of (A) IL-2, (B) TNFα, (C) IFNγ, (D) IL-22, (E) IL-17A, (F) IL-17F, (G) IL-6, (H) IL-9, (I) IL-10, (J) IL-4, (K) IL-5, and (L) IL-13 for T cells co-cultured with cDC1 (yellow-orange symbols), cDC2 (red symbols), DC3 (purple symbols) and pDC (blue symbols) of six donors (each donor with an individual symbol). Statistical analysis was performed in GraphPad Prism (V10) using 2way ANOVA for

grouped data with Dunnett's multiple comparisons tests as posthoc test (* $p < 0.05$, ** $p < 0.01$, *** $p < 0.001$, **** $p < 0.0001$).

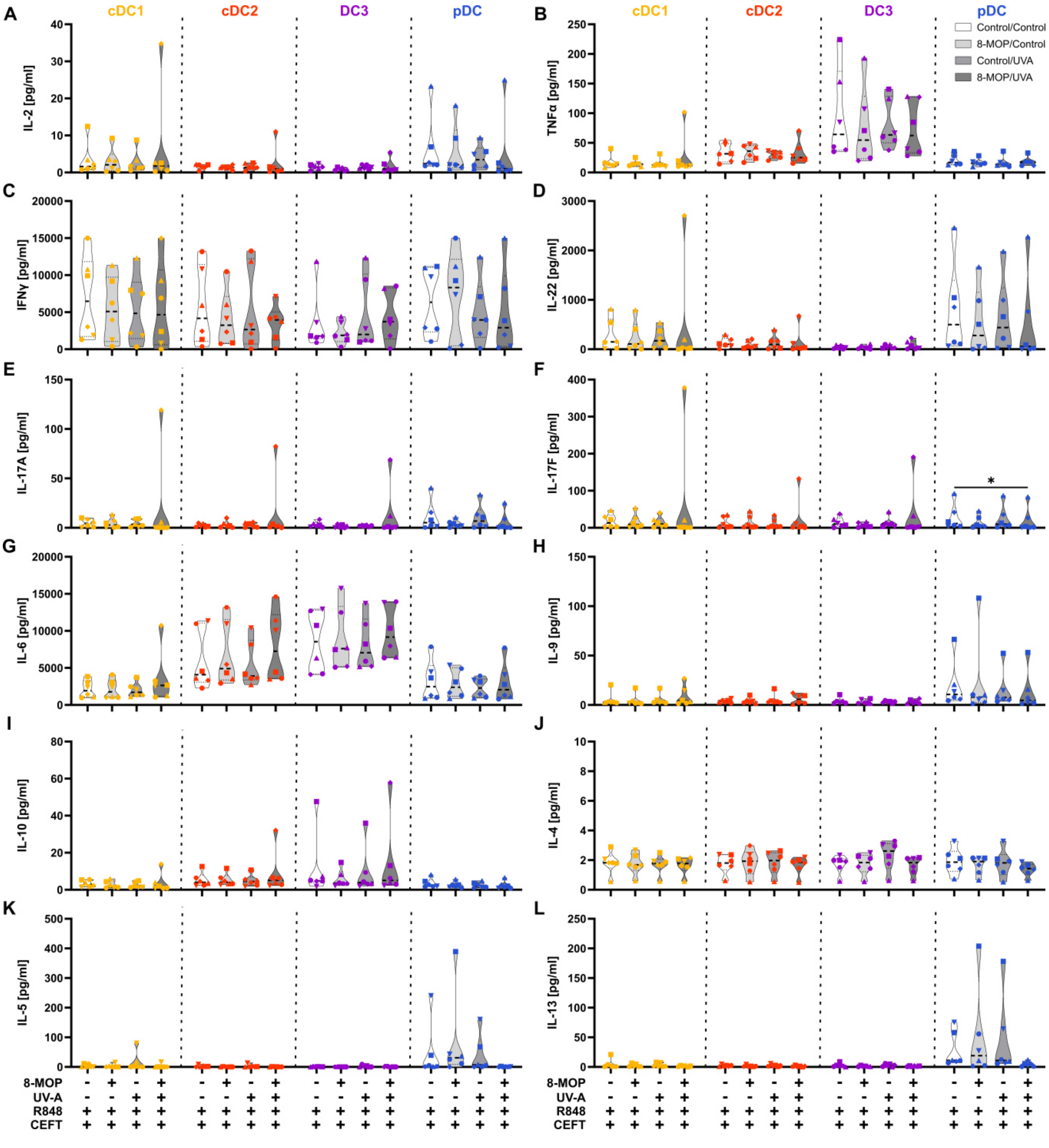

Supplementary Figure 8: Memory T cells restimulated by CEFT-loaded and R848-stimulated human DCs produce mainly IFNγ. Supernatants from DC:T cell co-cultures shown in Figure 7B were analyzed for the concentration of T cell-associated cytokines using the LEGENDplex Hu Th Cytokine Panel (BioLegend). Truncated violin plots show the concentrations of (A) IL-2, (B) TNFα, (C) IFNγ, (D) IL-22, (E) IL-17A, (F) IL-17F, (G) IL-6, (H) IL-9, (I) IL-10, (J) IL-4, (K) IL-5, and (L) IL-13 for T cells co-cultured with cDC1 (yellow-orange symbols), cDC2 (red symbols), DC3 (purple symbols) and pDC (blue symbols) of six donors (each donor with an individual symbol). Statistical analysis was performed in GraphPad Prism (V10) using 2way ANOVA for grouped data with

Dunnett's multiple comparisons tests as posthoc test (* $p < 0.05$, ** $p < 0.01$, *** $p < 0.001$, **** $p < 0.0001$).
